# Analysis of genome-wide DNA arrays reveals the genomic population structure and diversity in autochthonous Greek goat breeds

**DOI:** 10.1371/journal.pone.0226179

**Published:** 2019-12-12

**Authors:** S. Michailidou, G. Th. Tsangaris, A. Tzora, I. Skoufos, G. Banos, A. Argiriou, G. Arsenos

**Affiliations:** 1 Laboratory of Animal Husbandry, School of Veterinary Medicine, School of Health Sciences, Aristotle University of Thessaloniki, Thessaloniki, Greece; 2 Institute of Applied Biosciences, Center for Research and Technology Hellas, Thermi, Greece; 3 Proteomics Research Unit, Biomedical Research Foundation of the Academy of Athens, Athens, Greece; 4 School of Agriculture, Department of Agriculture, Division of Animal Production, University of Ioannina, Kostakioi Artas, Greece; 5 Scotland's Rural College and The Roslin Institute University of Edinburgh, Edinburgh, Scotland, United Kingdom; Universita degli Studi di Bologna, ITALY

## Abstract

Goats play an important role in the livestock sector in Greece. The national herd consists mainly of two indigenous breeds, the Eghoria and Skopelos. Here, we report the population structure and genomic profiles of these two native goat breeds using Illumina’s Goat SNP50 BeadChip. Moreover, we present a panel of candidate markers acquired using different genetic models for breed discrimination. Quality control on the initial dataset resulted in 48,841 SNPs kept for downstream analysis. Principal component and admixture analyses were applied to assess population structure. The rate of inbreeding within breed was evaluated based on the distribution of runs of homozygosity in the genome and respective coefficients, the genomic relationship matrix, the patterns of linkage disequilibrium, and the historic effective population size. Results showed that both breeds exhibit high levels of genetic diversity. Level of inbreeding between the two breeds estimated by the Wright’s fixation index F_ST_ was low (Fst = 0.04362), indicating the existence of a weak genetic differentiation between them. In addition, grouping of farms according to their geographical locations was observed. This study presents for the first time a genome-based analysis on the genetic structure of the two indigenous Greek goat breeds and identifies markers that can be potentially exploited in future selective breeding programs for traceability purposes, targeted genetic improvement schemes and conservation strategies.

## Introduction

The Greek national flock of goats is the largest in the European Union (E.U.), counting approximately 5 million heads [1]. These animals are classified into a wide range of farming systems; from semi-extensive low-input, traditional farms to large, semi-intensive, high producing and investing farms [2]. Most herds are traditionally reared in mountainous, semi-mountainous, lowland or insular areas, therefore are well adapted in different environmental conditions. For this reason, goat farming in Greece is preferred over other livestock systems in many marginal areas, because goats survive and produce in extreme environments and poor quality pastures (often including bushes and woody perennials), where other less versatile ruminants cannot.

Rearing of goats in Greece, traces back to Mycenaean times (about 1,200 B.C.); the first description on cheese making was reported by Homer and 500 years later, goats are found on silver coins in different Greek islands [3]. During the millennia of goat exploitation in Greece, many nuclei have evolved over time, forming different goat types, associated with their region of origin. Today, the vast majority of the Greek indigenous goats (90%) is comprised of the Eghoria breed, which is characterized by large variability in phenotypic characteristics. It is believed that Eghoria accounts for approximately 39 different types, which are mainly distinguished from their region of origin. The rest (10%), comprises of another autochthonous breed, the Skopelos (named after the homonymous island), which is a highly homogenous population of about 11,000 animals, as well as some foreign breeds (Damascus, Alpine and Saanen) and their crosses.

Herds of the Eghoria breed are found all over Greece, from isolated islands to inaccessible mountainous areas. This is a dual purpose breed, whose milk production ranges from 100 to 170 kg/lactation, depending on the rearing type. However, the genetic structure of this breed remains largely unknown, while it demonstrates large phenotypic, productive and reproductive variations. In contrast, Skopelos is a highly homogenous breed (based on phenotypic and productive traits), reared mainly at Northern Sporades island complex (Skopelos and Alonnisos islands). This breed has been intensively studied through national programs for the conservation of endangered indigenous breeds and for performance recordings, and has thus reached a milk yield of 225–260 kg/lactation. Historically, Skopelos and Eghoria breeds are assumed genetically distinct. The isolated geographical origin of Skopelos breed played a major role in supporting this premise. Yet, crossings between Eghoria and Skopelos breeds are usual, in order to increase milk performances in Eghoria and adaptability in more disadvantaged regions in Skopelos breed.

Unlike the cattle industry, where all Greek indigenous breeds are extinct or are in the verge of extinction due to the advent of more productive, high yielding animals (eg. Holstein-Friesian cattle), many indigenous breeds of small ruminants are still reared in Greece. However, during the last years, many goat herds are transforming, creating nuclei of crossbred animals (indigenous x imported purebreds). This diverse national herd has only been studied with few nuclear molecular markers, as single nucleotide polymorphisms (SNPs) [4, 5], amplified fragment length polymorphisms (AFLPs) [6] or mitochondrial DNA analysis [7]. During the last few years, the development of a genotyping microarray by the International Goat Genome Consortium (IGGC) [8], alongside with the availability of a goat reference genome [9, 10], have accelerated the understanding on domestication and genetic diversity in goat breeds, eliminating the errors introduced by the use of traditional genetic approaches.

Despite having the largest goat population in the E.U., Greece ranks in the 5^th^ place in E.U. in total milk yield, after France, Czech Republic, Spain and Estonia, based on official data recorded for 2015 [1]. This is explained by the low productivity per doe, derived from the traditional and low input management systems applied in goat farming [2]. Although goat population in Greece is large compared to other livestock species, goat sector has a small impact in food industry in Greece. This reflects in analogous poor management systems and budgets, resulting in many cases in herds lacking rudimentary essentials like properly written and updated herd-books. However, intensive farming systems in Greece are more vulnerable, entailing higher levels of uncertainty due to increases in feed costs and changes in environmental conditions [2]. Thus, in order to develop and render Greek goat farming sustainable, actions should be taken to accurately predict the genomic breeding value of an individual. Therefore, studying the genetic structure of a breed and understanding its genetic background will facilitate the genetic improvement in a population, as it is easier to monitor significant genes correlated with productivity, disease resistance or survival traits and therefore, easier to determine resilient individuals. To that end, the AdaptMap project (http://www.goatadaptmap.org/) is a valuable initiative led by international scientific groups that offers genotyping data of 130 breeds reared worldwide, so that can be used to monitor gene flow, migration events and genomic admixture levels with local or cosmopolitan breeds [11–16]. Such comparisons will help on the creation and preservation of purebred nuclei, which is of paramount importance as selected animals can be used in targeted breeding schemes utilizing the unique genetic resources.

Genomic characterization and protection of breeds has been extensively discussed in the past as a means to design breeding programs and future conservation strategies [17–19]. Moreover, breed traceability is a means to protect and valorize particular food products, and enhance consumer confidence towards foods of particular animal origin [20]. Yet, purebreds should be used with caution in selective breeding schemes, since inbreeding must be controlled to limit the potential impact of deleterious alleles and inbreeding depression on animal traits, and the loss of genetic variance [21, 22].

In the present study, we report a comprehensive genome-wide analysis of the population structure and genomic diversity of two Greek goat breeds, based on the genotypes generated by the Goat SNP50 BeadChip. Furthermore, we aim to identify genomic regions that could be used as potential markers to distinguish breeds and be further used in breed identification and conservation strategies.

## Materials and methods

### Ethics statement

All applicable international, national, and/or institutional guidelines for the care and use of animals were followed. In accordance to the 2010/63/EU guide and the adoption of the Law L276/33/20.10.2010 by the Greek Government (Law 106/vol A/30.04.2013), ethical approval is not required in our study. Blood sampling was conducted by veterinarians and/or under veterinarian supervision for routine veterinary care. All samples and data in our study were collected under the consent of the breeders.

### Animal sampling

A total of 72 samples of two Greek domestic goat breeds (32 of Eghoria and 40 of Skopelos) were collected from 6 farms located in Central and Northern Greece. All animals were reared under semi-intensive conditions. Four milliliters of blood were collected from the jugular vein of each female goat in tubes containing EDTA as anticoagulant and stored in a freezer (-20°C) until further use in the laboratory. Breed discrimination was based on the available pedigree, morphology and productive trait records. Farm locations are indicated in a geographical map of Greece (S1 Fig) which was created in R [23] using the packages ‘maps’ [24], ‘rworldmap’ [25] and ‘rwordxtra’ [26]. Details on farms locations and number of animals sampled per farm and breed are presented on S1 Table.

### DNA extraction and genotyping

DNA was extracted from blood samples using the Nucleospin Blood Quick Pure kit (Macherey-Nagel, Germany) according to the manufacturer’s protocol. DNA integrity was assessed by electrophoresis on a 0.8% TAE agarose gel. DNA concentration was quantified with the Qubit® dsDNA BR assay kit (Qubit 2.0 Fluorometer, Invitrogen, Carlsbad, CA, USA). All animals were genotyped using Illumina’s Goat SNP50 BeadChip containing 53,347 SNPs. DNA samples were dried down in 1 mM Tris-EDTA and 400 ng of each sample was sent to GeneSeek (Neogen Corporation, UK) for genotyping on an Illumina’s iScan platform. Imaging and data generation were performed with the Illumina’s iScan system.

### Data analysis

#### Sample and SNP quality control

Genotypes were generated and pre-processed by the iScan system based on an automated genotype calling feature by Illumina’s GenomeStudio software (v.1.9.4). During this step a preliminary quality control on individuals (exclusion or inclusion of samples) and basic statistics for each SNP are generated e.g. call rate, allele frequencies. Filtering of samples was performed based on the call rate for each sample (call rate>0.9) generated by Illumina’s GenomeStudio software, as well as using the—*mind* command in PLINK v1.90 [27]. Chromosomal coordinates for each SNP were mapped to the updated goat reference ARS1 assembly [9].

PLINK v1.90 was used to generate summary statistics and conduct exact tests for deviation from Hardy–Weinberg equilibrium (HWE). Quality control and SNP filtering were performed using PLINK v1.90 [27] by filtering out SNPs with minor allele frequency (MAF) <1%, call rate <0.98, HWE p-value ≤1,0E-6, those lacking genomic location and sex-linked SNPs, since they exhibit lower mutation rates and a direct comparison of the overall diversity between the X chromosome and autosomes is difficult [28, 29].

#### Population structure analysis

Assessment of the genetic structure of individuals was performed in autosomal filtered SNPs using the ADMIXTURE v1.3 software [30], assuming a number of subpopulations (K) ranging from 2 to 5 and visualized using R plots. The optimal number of K value was determined as the one having the lowest cross-validation (CV) error. Population structure was also examined by principal component analysis (PCA) in autosomal SNPs; PLINK was used for the generation of eigenvectors and eigenvalues which were visualized with the GENESIS software [31] demonstrating the relationship between PC1 and PC2.

Genetic structure of the two Greek goat breeds was also analyzed including data from 45 breeds reared worldwide, in order to assess whether admixture with cosmopolitan breeds has occurred in Greek breeds. Genotypes were obtained from the AdaptMap project [12] and are available in https://doi.org/10.5061/dryad.v8g21pt. Selection of the 45 goat breeds was based on the transit of goats from their putative center of domestication to Europe, both from the Danubian corridor and the Mediterranean basin. Thus, data from 29 breeds from Europe, 3 breeds from Near East, 9 breeds from Northern Africa and 4 breeds from eastern Asia were selected for comparison purposes with Greek data. Quality control and SNP filtering were also performed using PLINK v1.90, by filtering out SNPs in autosomes with minor allele frequency (MAF) <1% and call rate <0.98. Population substructure among the 47 goat populations using ADMIXTURE software was conducted assuming a number of K from 2 to 50. Visualizations of ancestry coefficients estimated with ADMIXTURE software were performed using the BITE R package [32].

To further investigate historical gene flow between the populations analyzed, we used Treemix software v.1.12 [33]. Maximum likelihood trees were constructed for different migration events, starting from *m0* (no migration event) to *m15*, by grouping SNPs in windows of 200. To increase robustness of analysis, we applied three replicates per migration event. The percentage of fraction of variance explained for each *m*, the log-likelihood value for each *m* and the residuals from the fit of the model among individuals were used to determine and visualize the most predictive model (*m*_*best*_). Then, nodes robustness was estimated for the *m*_*best*_ by running 100 bootstrap replicates using the *Treemix_boostrap*.*sh* script and plotted using the *treemix*.*bootstrap* R function, both implemented in BITE R package [32].

#### Genetic diversity indices and inbreeding levels

Observed (Ho) and expected (He) heterozygosities were calculated for each breed separately to measure the genetic diversity within breed using ARLEQUIN 3.5.2.2 software [34], by applying a correction on the number of usable SNPs proposed by Colli et al. [14]. Wright’s inbreeding coefficient F_IS_ (Individual within Subpopulation) was calculated per animal using PLINK. Genomic inbreeding was also calculated following Van Raden [35]; computation of inbreeding values were assessed from the diagonal of the genomic relationship matrix, denoted as F_GRM_. Estimates of inbreeding coefficients were calculated from autosomes, for each animal separately, using the GCTA program [36] starting from the PLINK binary PED files (.bed, .fam, .bim). Wright’s pairwise F_ST_ value was calculated using ARLEQUIN software to test inbreeding levels and genetic distance between breeds.

#### Runs of homozygosity

Runs of homozygosity (ROH) were defined in all autosomes to assess inbreeding levels for all animals, in each breed separately, and categorized based on their length and chromosome using PLINK v1.90. ROHs were calculated with the ‘Runs of homozygosity’ function in PLINK software with adjusted parameters for the total length of ROHs (≥1Mb), the variants in the scanning window (n = 15), the number of heterozygous SNP allowed (n = 1) and the number of missing calls (n = 1) to estimate homozygosity. The percentage of ROHs per chromosome was calculated as proposed by Al-Mamun et al. [37], as follows:

$$Average\;percentage\;of\;ROH\;per\;chromosome=\frac{\sum ROHs\;in\;Mbp\;per\;chromosome}{N*Chromosome\;length\;(Mbp)}*100$$

where N is the number of animals that had a ROH in that chromosome.

Genomic inbreeding coefficient based on ROHs (F_ROH_) was calculated for each animal from the sum of ROH lengths, divided by the total length of the autosomal genome (kb) covered by SNPs as proposed by McQuillan et al. [38]. Inbreeding coefficients based on ROHs were calculated for four bins, grouped according to the length of ROHs i) all ROHs (F_ROH_), ii) <10 Mb (F_ROH <10Mb_), iii) 10–20 Mb (F_ROH 10-20Mb_) and iv) >20 Mb (F_ROH >20Mb_). In all cases, coefficients were calculated separately for each animal and then averaged within breed.

ROH frequencies were calculated for each breed and for six different length categories (<3 Mbp, 3–5 Mbp, 5–10 Mbp, 10–20 Mbp, 20–30 Mbp, >30 Mbp), using the same classification reported by the AdaptMap project for comparison purposes [12]. ROH frequencies were plotted for each length category and for each breed separately, by summarizing all ROHs and averaged against the total length of ROHs (in Mbp).

To identify SNPs that are present inside ROHs the R package ‘detectRUNS’ was used [39]. The genome-wide occurrence of SNPs in ROHs was expressed as the proportion (%) of times each SNP fall inside the defined ROHs, plotted against SNP position per chromosome, for each breed separately.

#### Linkage disequilibrium analysis

Linkage disequilibrium (LD) was tested for each breed separately to examine recombination of linked SNPs using PLINK v1.90 with default parameters; SNPs included in this step spanned a distance from 0.001 to 1 Mb. The squared correlation coefficient (r^2^) curve was estimated by determining the nonlinear least squares fit line using the *nls* function in R. The r^2^ coefficient was used instead of D’ as a more reliable measurement in studies with small sample sizes and more useful in predicting the power of association mapping [40].

#### Effective population size analysis

Effective population size (Ne) was estimated separately for each breed, using SNeP v.1.1 [41]. Ne estimates at different generations were based on linkage disequilibrium using the formula suggested by Corbin and co-authors [42]. Estimated effective population size was plotted over the last 150 and 1,000 generations to assess relevant diversity trends.

#### Discriminatory SNPs among breeds

In order to identify regions putatively under selection and SNPs that can be used for the discrimination of breeds, three different approaches were carried out:

- Calculation of Fst for each marker: Fst value for each SNP was calculated with the—*fst* -*within* command in PLINK v1.90, using the method introduced by Weir and Cockerham (W&C) [43]. SNPs at a threshold corresponding to the 0.995 percentile of the total distribution were acquired for gene annotation. Manhattan plots demonstrating the Fst value for each SNP were constructed using the qqman R package [44].
- Identification of discriminatory SNPs for breed assignment using the Toolbox for Ranking and Evaluation of SNPs (TRES) software [45]. Evaluation of SNPs was performed by comparing SNPs obtained from all available methods; Delta [46], Wright’s pairwise Fst [47], and Informativeness for Assignment [48]. From each analysis, the top 200 SNPs were required, and only common SNPs among methodologies were further evaluated. Using the TRES software two methodologies were followed:
  - Identification of discriminatory SNPs was performed using the whole dataset of animals per breed, denoted as ‘TRES_all’.
  - Identification of discriminatory SNPs was performed by randomly splitting the dataset into training and test populations (using the—*awk srand(n)* function), denoted as ‘TRES_tt’.

Consensus SNPs among the three methods (Fst, TRES_all, TRES_tt) were obtained and evaluated using GeneClass2 [49]. SNPs per methodology were submitted to Venny 2.1 [50] to depict common and/or shared SNPs. For the evaluation of discriminatory SNPs, two approaches were followed; assignment or exclusion of individuals based on the discriminatory SNPs and detection of first generation migrants using the likelihood (L) estimation calculated from L_home/L_max [51]. In both approaches, SNPs were evaluated using frequency-based [52] and Bayesian [53] criteria. In all cases, Monte Carlo resampling was enabled, using the simulation algorithm proposed by Paetkau et al. [51] with a number of simulated individuals of 1,000 and a type I error (alpha) threshold of 0,001.

Discriminatory SNPs obtained from all methods were annotated on the ARS1 assembly [9] using the Genome Data Viewer from National Center for Biotechnology Information (NCBI). Annotation was performed to reveal genes or nearby genes (within ±100kb) from the positions of identified SNPs that might indicate signatures of selection for the two breeds.

## Results

### Sample and SNP filtration, basic descriptive statistics

No sample was excluded from further analysis; mean call rate for the 72 samples was 0.974. Quality control of SNPs was performed by filtering out non-informative SNPs for minor allele frequency (MAF), call rate (call frequency) and HWE. As such, 4,506 SNPs were excluded, resulting in 48,841 SNPs kept for downstream analysis (S2 Table). Genotyping data (.ped and .map files) are publicly accessible via Zenodo database (https://zenodo.org/record/3073175#.XOPaAthRWHt).

### Population structure analysis

**Fig 1:**
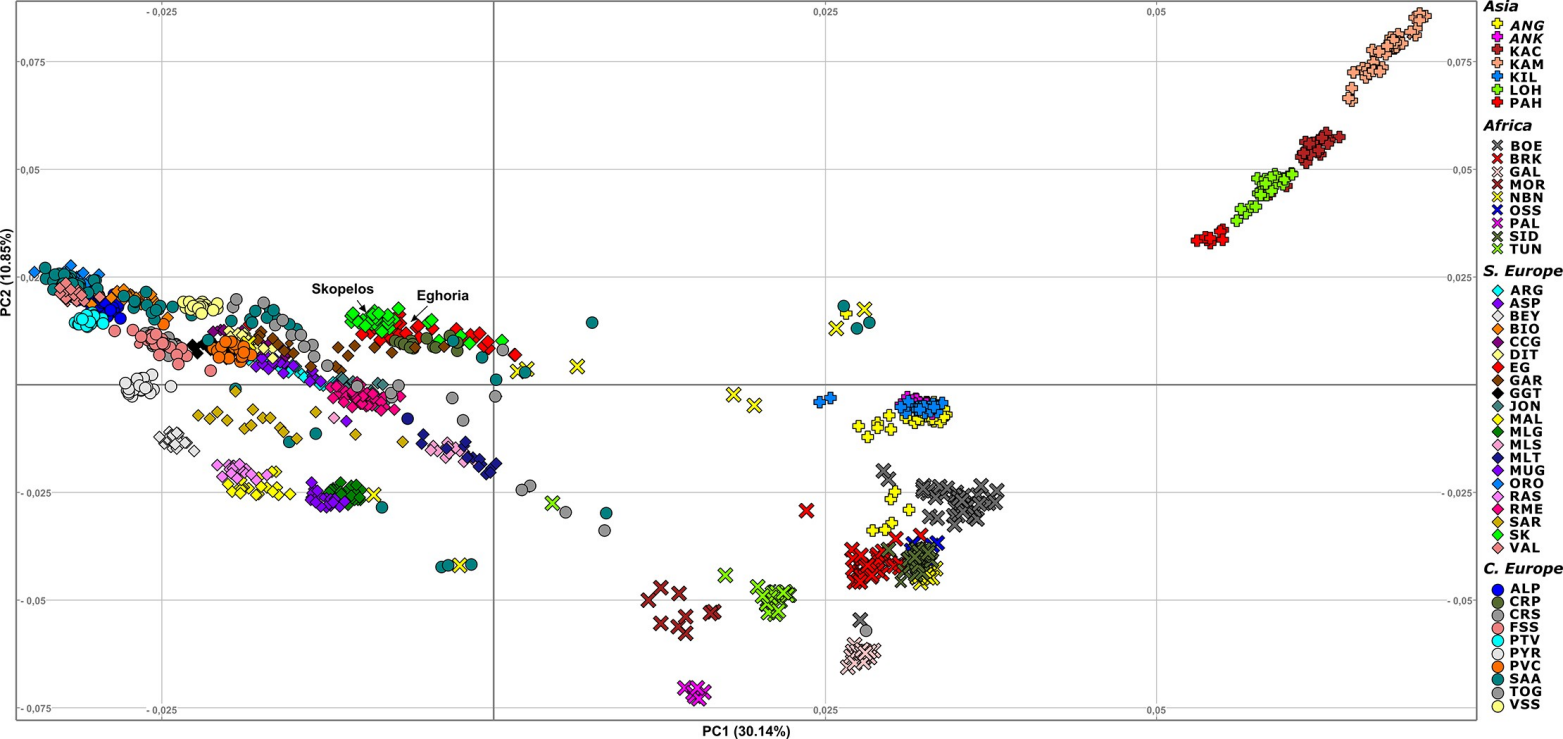
Principal component analysis of the first two axes in 1,212 goat samples from 47 breeds. EG: Eghoria; SK: Skopelos; ALP: Alpine; ANG: Angora; ANK: Ankara; ARG: Argentata; ASP: Aspromontana; BEY: Bermeya; BIO: Bionda dell'Adamello; BOE: Boer; BRK: Barki; CCG: Ciociara Grigia; CRP: Carpatian; CRS: Corse; DIT: Di Teramo; FSS: Fosses; GAL: Galla; GAR: Garganica; GGT: Girgentana; JON: Jonica; KAC: Kachan; KAM: Kamori; KIL: Kil; LOH: Lohri; MAL: Mallorquina; MLG: Malaguena; MLS: Maltese Sarda; MLT: Maltese; MOR: Moroccan goat; MUG: Murciano-Granadina; NBN: Nubian; ORO: Orobica; OSS: Oasis; PAH: Pahari; PAL: Palmera; PTV: Poitevine; PVC: Provencale; PYR: Pyrenean; RAS: Blanca de Rasquera; RME: Rossa Mediterranea; SAA: Saanen; SAR: Sarda; SID: Saidi; TOG: Toggenburg; TUN: Tunisian; VAL: Valdostana; VSS: Valpassiria.

**Fig 2:**
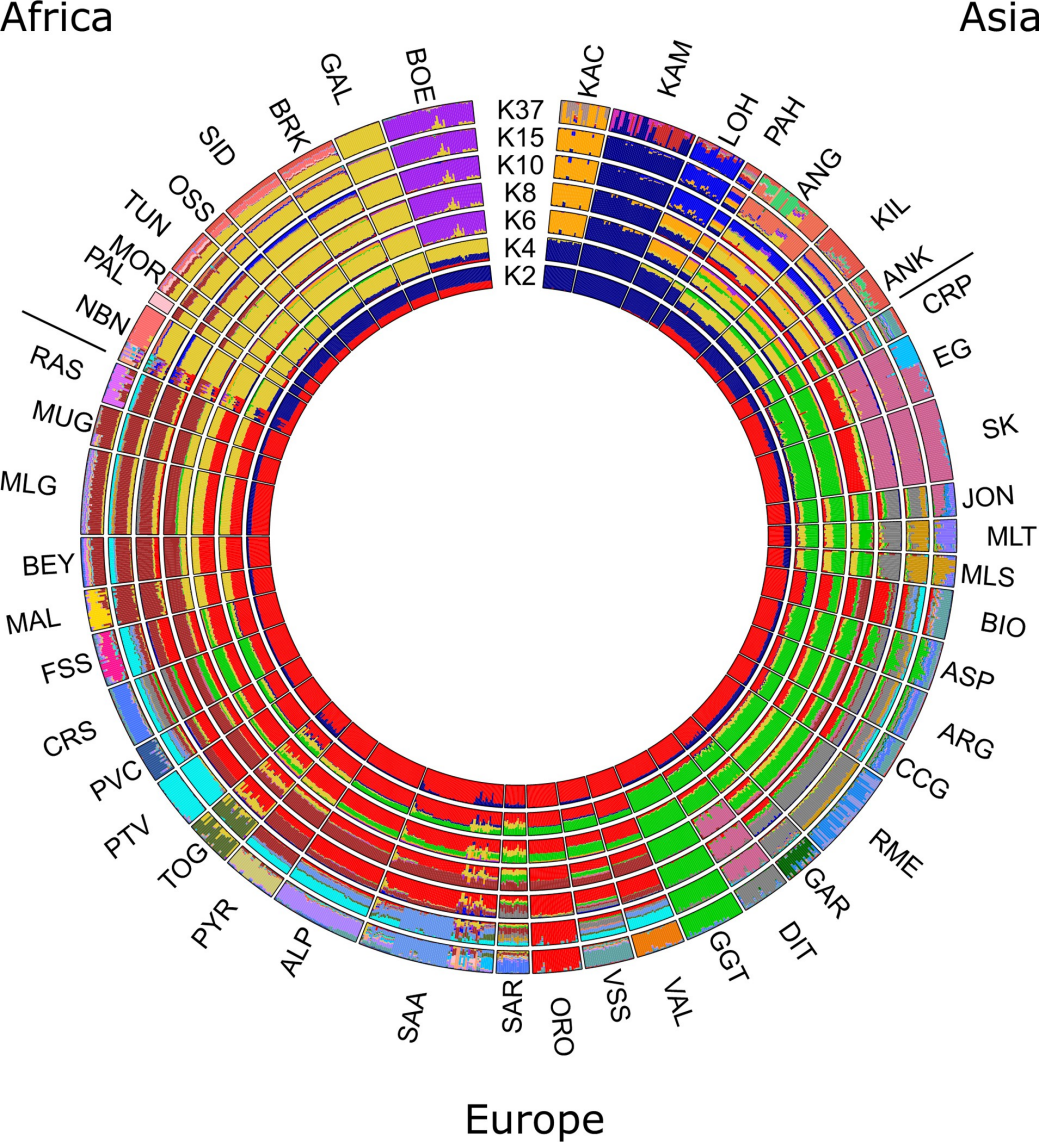
Circular representation of Admixture analysis at K = 2, 4, 6, 8, 10, 15 and 37 for 1,212 goat samples from 47 breeds. Each individual is presented by a vertical bar. Different colors indicate different clustering groups. EG: Eghoria; SK: Skopelos; ALP: Alpine; ANG: Angora; ANK: Ankara; ARG: Argentata; ASP: Aspromontana; BEY: Bermeya; BIO: Bionda dell'Adamello; BOE: Boer; BRK: Barki; CCG: Ciociara Grigia; CRP: Carpatian; CRS: Corse; DIT: Di Teramo; FSS: Fosses; GAL: Galla; GAR: Garganica; GGT: Girgentana; JON: Jonica; KAC: Kachan; KAM: Kamori; KIL: Kil; LOH: Lohri; MAL: Mallorquina; MLG: Malaguena; MLS: Maltese Sarda; MLT: Maltese; MOR: Moroccan goat; MUG: Murciano-Granadina; NBN: Nubian; ORO: Orobica; OSS: Oasis; PAH: Pahari; PAL: Palmera; PTV: Poitevine; PVC: Provencale; PYR: Pyrenean; RAS: Blanca de Rasquera; RME: Rossa Mediterranea; SAA: Saanen; SAR: Sarda; SID: Saidi; TOG: Toggenburg; TUN: Tunisian; VAL: Valdostana; VSS: Valpassiria.

The genetic structure among the two Greek breeds and the 45 selected breeds reared worldwide, assessed from PCA, revealed three major clusters; one enclosing European breeds, a second enclosing only breeds from Pakistan (Central Asia) and a third looser cluster consisting of breeds from Asia and Africa (Fig 1). Results showed that Greek breeds cluster together with European breeds and are found in greater distance from the other two clusters. From the breeds that clustered near Greek breeds, PCA revealed that Eghoria and Skopelos grouped closer to the Carpatian breed (CRP), as well as to Italian breeds, such as Garganica (GAR), Rossa Mediterranea (RME) and Jonica (JON). Admixture analysis revealed that Greek breeds maintain a distinct genetic profile compared to other European breeds, although cosmopolitan breeds like Alpine and Saanen are reared form many decades in Greece (Fig 2). The lowest CV error for the 47 breeds was acquired for K = 37.

**Fig 3:**
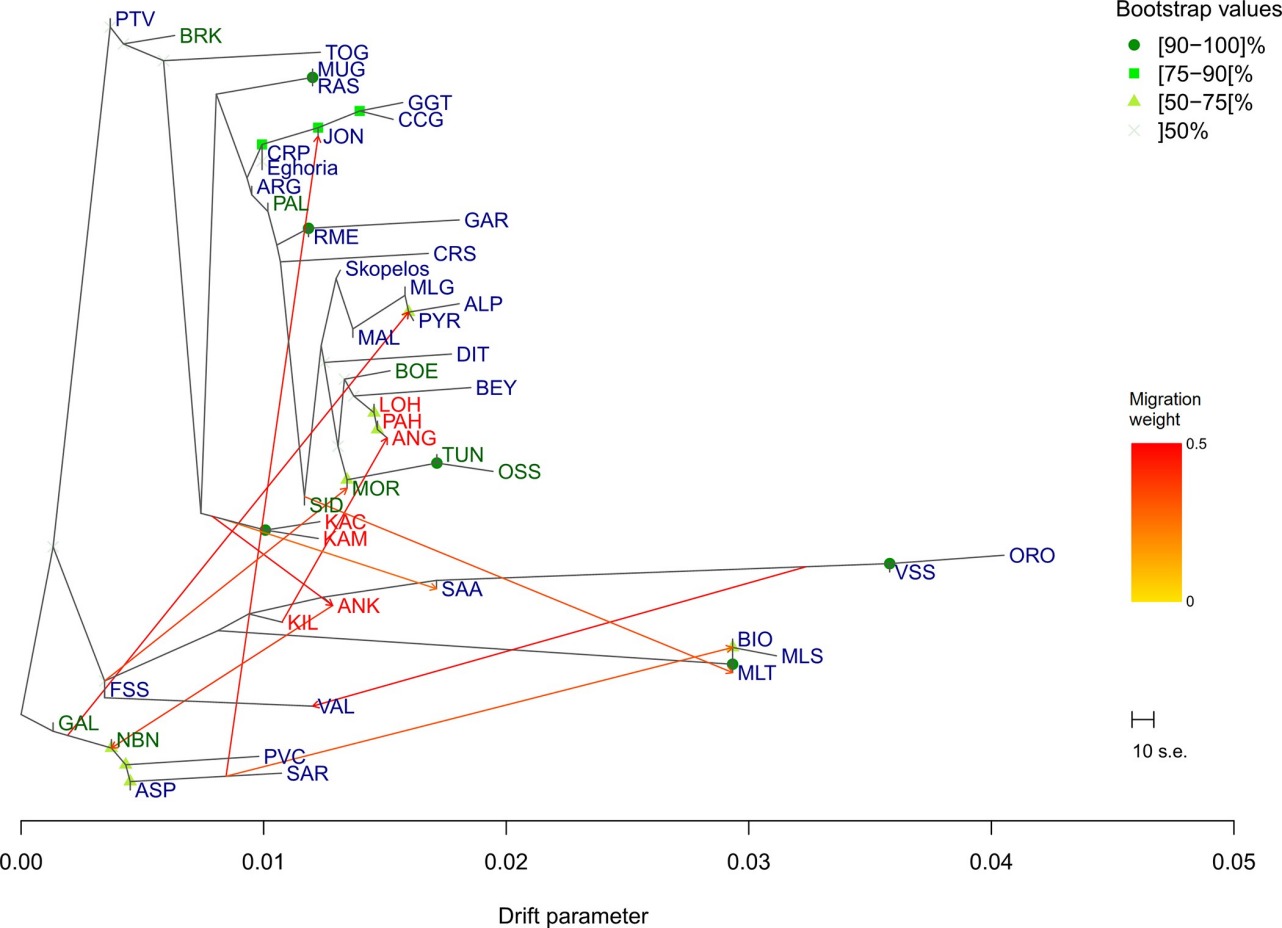
Treemix analysis with 10 migration events. Nodes robustness was estimated with 100 bootstrap replicates. Bootstrap values below 50 are not shown. Migration edges are colored according to their migration weight. Breeds are colored according to their geographical origin (blue for European, red for Asian and green for African breeds). ALP: Alpine; ANG: Angora; ANK: Ankara; ARG: Argentata; ASP: Aspromontana; BEY: Bermeya; BIO: Bionda dell'Adamello; BOE: Boer; BRK: Barki; CCG: Ciociara Grigia; CRP: Carpatian; CRS: Corse; DIT: Di Teramo; FSS: Fosses; GAL: Galla; GAR: Garganica; GGT: Girgentana; JON: Jonica; KAC: Kachan; KAM: Kamori; KIL: Kil; LOH: Lohri; MAL: Mallorquina; MLG: Malaguena; MLS: Maltese Sarda; MLT: Maltese; MOR: Moroccan goat; MUG: Murciano-Granadina; NBN: Nubian; ORO: Orobica; OSS: Oasis; PAH: Pahari; PAL: Palmera; PTV: Poitevine; PVC: Provencale; PYR: Pyrenean; RAS: Blanca de Rasquera; RME: Rossa Mediterranea; SAA: Saanen; SAR: Sarda; SID: Saidi; TOG: Toggenburg; TUN: Tunisian; VAL: Valdostana; VSS: Valpassiria.

Treemix analysis verified ADMIXTURE and PCA results, since no gene flow between Alpine and Saanen and Greek breeds is observed at *m10* (Fig 3). Overall, the fraction of variance explained in our dataset ranged from 89.49% (*m0*) to 95.03% (*m15*). The model with the 10 migration edges was chosen as the most predictive model since it explains 93.52% of the variance, and all migration events thereafter (*m11* to *m15*) did not account for much more variance (S2 Fig). Moreover, identification of the populations that are not well-modeled at *m10* presented only a few pairs of populations, mostly of Italian origin, with high standard error that are not well explained, thus are candidates for admixture events (S3 Fig). The consensus tree obtained from the 100 replicates for the Greek breeds showed that their nodes were supported by bootstrap values below 50. Eghoria and Skopelos breeds were located in separate nodes, grouped together however with Europeans breeds. No major migration event was observed for the Greek breeds at *m10*. Skopelos breed was found as a surrounding population (together with MLG, MAL and ALP) to a high-weighted edge that links Galla breed (GAL) from Kenya to Pyrenean breed (PYR). Eghoria breed was located in a node enclosing Carpatian (CRP), Jonica (JON), Girgentana (GGT) and Ciociara Grigia (CCG) breeds. This clade was found to be linked with a high-weighted edge originating from Italian breeds Aspromontana (ASP) and Sarda (SAR).

**Fig 4:**
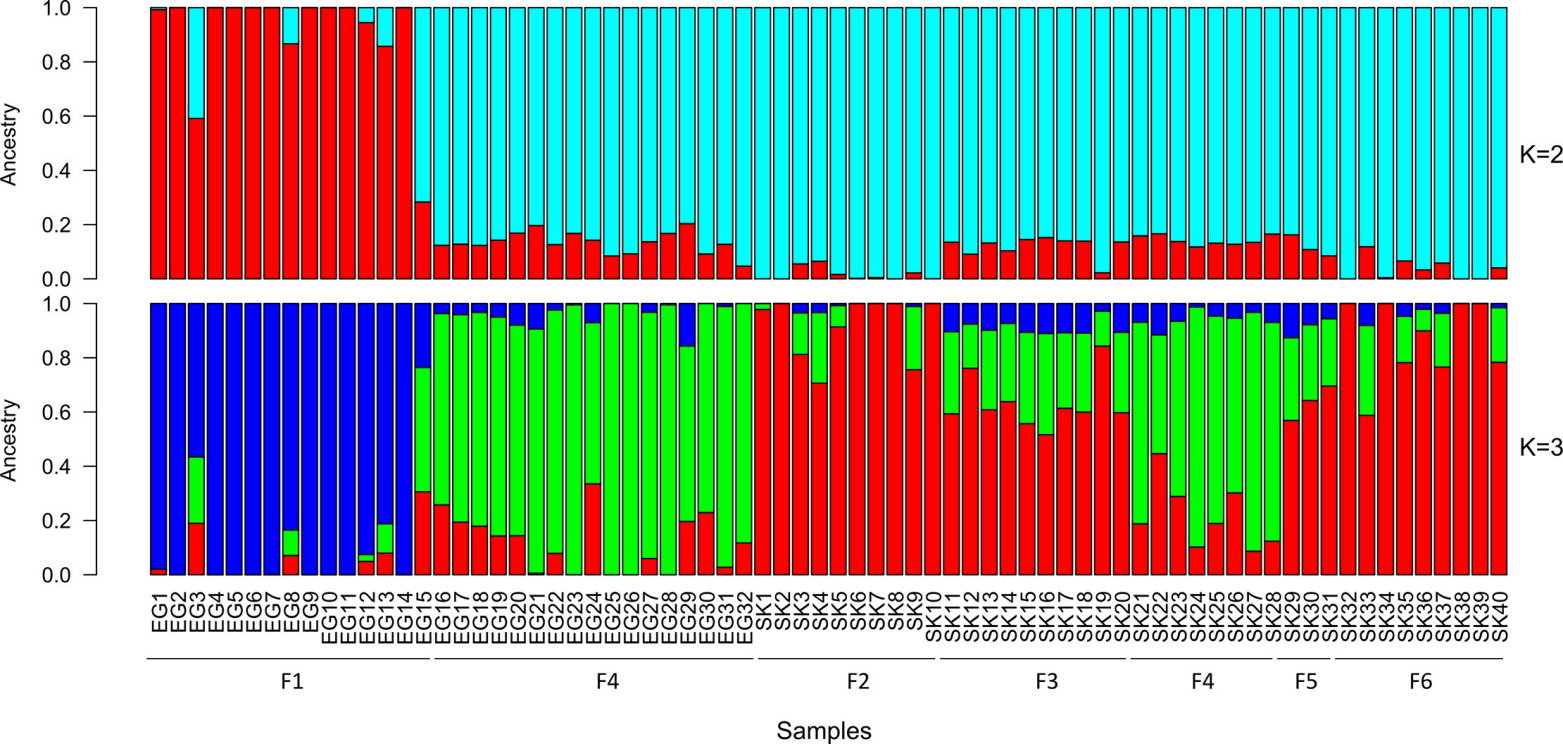
Admixture analysis at K = 2 and 3 for the 48,841 autosomal SNPs in Greek breeds. Each individual is represented by a vertical bar. Different colors indicate different clustering groups. F1: farm 1; F2: farm 2; F3: farm 3; F4: farm 4; F5: farm 5; F6: farm 6. SNPs: Single nucleotide polymorphisms.

Population substructure was also assessed exclusively in Greek goats, using both ADMIXTURE and PCA analysis in autosomal filtered SNPs in order to evaluate purebreds and detect possible crossbreds. Population structure was investigated assuming a number of K from 2 to 5. Cross validation error was the lowest for K = 3 (0.63661), indicating the most likely number of different sub-populations represented in the 72 samples [30]. At K = 2 the first group that differentiated consisted of individuals of the Eghoria breed (samples EG1 to EG14), all originating from the same farm (farm 1 in S1 Fig) located in a geographical isolated region in the Pindus mountain range (Fig 4). At K = 3, this nucleus remained differentiated from the others and two additional clusters were formed. Individuals of Skopelos breed exhibited a more uniform profile, including however eight samples (SK21 to SK28) originating from the municipality of Thessaloniki (farm 4 in S1 Fig) that had a somewhat different genetic profile. Notably, this particular nucleus of Skopelos individuals had the same genetic profile with many samples of Eghoria breed (samples EG16 to EG32), originating from the same farm, too. At K = 4 the clusters that were formed were similar to those obtained at K = 3. The main difference was that the classification of individuals of farm 1 started to disappear progressively. At the highest K value of 5, cross-validation error reached 0.67373 and a weaker population substructure was observed, maintaining however the main trend in the clustering profile. From the aspect of farms, the mean component values per farm and K were calculated to reveal potential geographic differentiation patterns; similar clustering profiles were acquired with farm 1 being the most differentiated, followed by farm 4 (S4 Fig).

**Fig 5:**
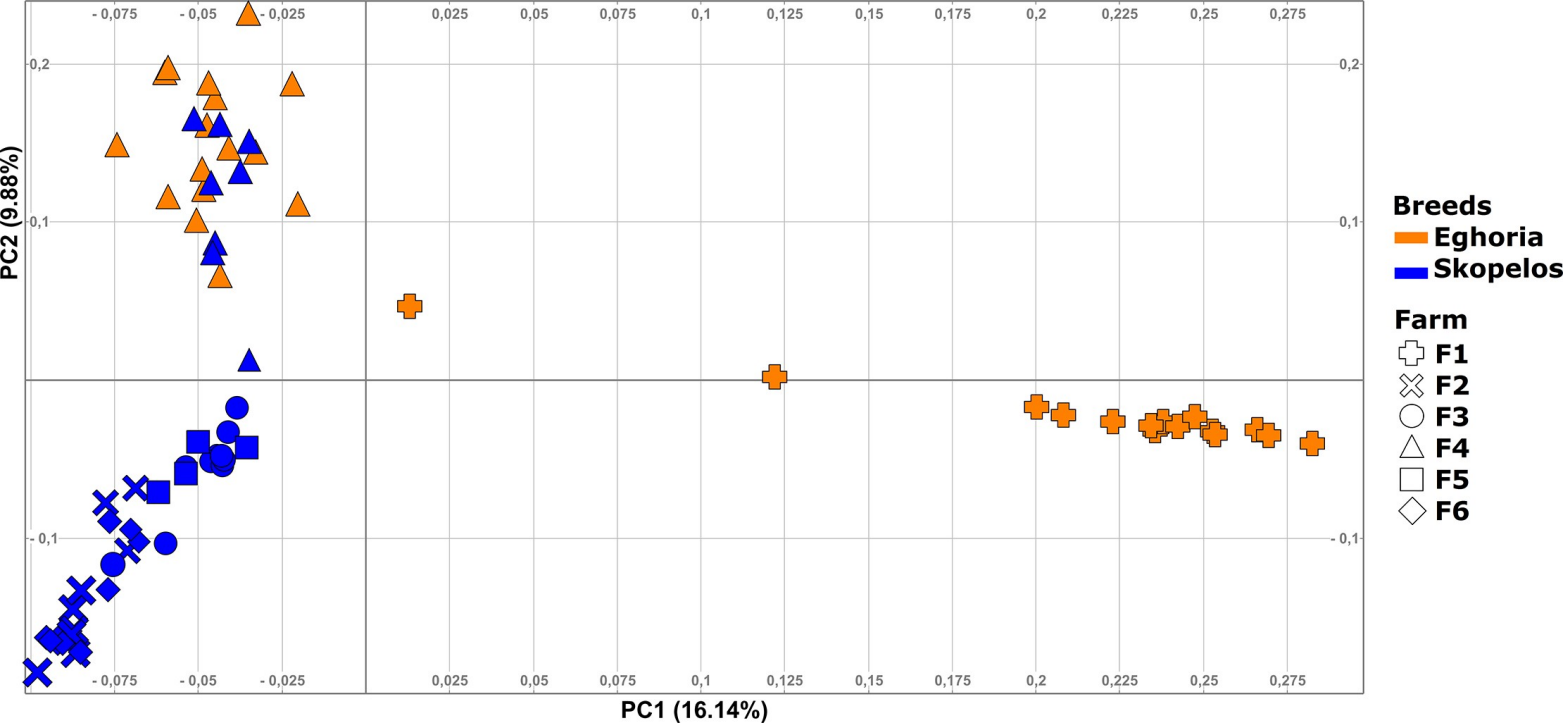
Principal component analysis of the first two axes in 72 goat samples using the 48,841 SNPs. SNPs: Single nucleotide polymorphisms.

PCA analysis in the 48,841 autosomal SNPs revealed the same clustering profile with ADMIXTURE analysis. The first two principal components (PC1 and PC2) explained 26.02% of the total genetic variation. In particular, three distinct clusters were formed, associated with region of origin (Fig 5). Analysis of principal components showed that Eghoria breed possesses higher levels of genetic variation, compared to Skopelos breed. Individuals of the Eghoria breed originating from farm 1 formed a distinct cluster in a greater distance from all the remaining samples, which was also the case in clustering at K = 2. From the other two clusters, the one consisted only of Skopelos individuals who are reared in their region of origin (farms 2, 3, 5, 6 in S1 Fig) and the third, the one with the admixed population, consisted of samples from both breeds, which were reared in the same farm. Considering that the admixed animals of Skopelos breed could introduce errors in the estimation of genetic heterozygosity and inbreeding indices, they were excluded from downstream analysis.

### Within and between breed genetic diversity

After the exclusion of admixed animals, the new dataset consisted of 64 animals in total (32 female goats for each breed). From the 48,841 quality-filtered SNPs, the percentage of within-breed polymorphic loci was greater than 95% for both breeds (Table 1). The number of polymorphic loci was 46,608 and 46,732 SNPs for Eghoria and Skopelos breeds, respectively. Even though only the Skopelos breed had been used for the validation of Goat SNP50 BeadChip [8], the Eghoria breed also maintained high levels of polymorphic loci. According to Ho and He values, Eghoria breed presented higher within-breed level of genetic diversity than Skopelos, yet differences between breeds were not substantial. Mean He values were 0.405±0.110 and 0.394±0.120 for Eghoria and Skopelos breeds, respectively. Intra-population nucleotide diversity estimated from π values, was quite similar between breeds (0.404 and 0.392 for Eghoria and Skopelos breeds, respectively).

**Table 1:** Genetic diversity within breeds derived from individuals per breed (N), as measured by the number (N_P_) and percentage of polymorphic loci (N_P%_), observed (Ho) and expected (He) heterozygosity with standard deviation (±sd) and nucleotide diversity per breed (π), for the 48,841 filtered single nucleotide polymorphisms (SNPs).

Breed	N	N_P_	N_P%_	Ho (±sd)	He (±sd)	π
**Eghoria**	32	46,608	95.42%	0.395±0.132	0.405±0.110	0.404
**Skopelos**	32	46,732	95.68%	0.392±0.140	0.394±0.120	0.392

Different estimates of inbreeding coefficients were calculated from the SNP genotyping data. Wright’s inbreeding coefficient F_IS_ was calculated separately for each individual; both breeds presented positive mean F_IS_ values (0.0312 and 0.0376 for the Eghoria and Skopelos breeds, respectively) indicating more homozygotes in the studied population than expected (Table 2). Lower inbreeding coefficient values were obtained for the Skopelos breed compared to Eghoria, in the other two indices studied (F_GRM_ and F_ROH_) (Table 2). The mean value of the genomic estimator F_GRM_ was positive for both breeds, but very close to 0 (0.0452 for Eghoria and 0.0211 for Skopelos breeds), indicating that the variance is low. Inbreeding coefficient based on all ROHs revealed higher levels of autozygosity in Eghoria (F_ROH_ = 0.0680) than in Skopelos breed (F_ROH_ = 0.0287). Among the different F_ROH_ bins, F_ROH >20Mb_ expressed the highest values for both breeds. Moreover, at small lengths, where very short and common ROHs are located due to LD [54], F_ROH <10Mb_ presented very low values in both breeds. Overall, differences on levels of inbreeding reflected by F_IS_, F_ROH_ and F_GRM_ were quite modest; similar levels of inbreeding were found between the two breeds. Genetic differentiation of breeds and the level of their relatedness, indicated by pairwise F_ST_ values was low (0.04362).

**Table 2:** Average inbreeding coefficients and standard errors (SE) per breed, estimated by Wright’s inbreeding coefficient (F_IS_), genomic relationship matrices inbreeding coefficient (F_GRM_) and derived from runs of homozygosity inbreeding coefficient (F_ROH_) for different length categories.

				F_ROH_ (±SE)		
Breed	F_IS_ (±SE)	F_GRM_ (±SE)	F_ROH_ (±SE)	F_ROH<10Mb_ (±SE)	F_ROH 10-20Mb_ (±SE)	F_ROH >20Mb_ (±SE)
**Eghoria**	0.0312 (±0.0151)	0.0452 (±0.0146)	0.0680 (±0.0112)	0.0009 (±0.0000)	0.0000 (±0.0000)	0.0712 (±0.0112)
**Skopelos**	0.0376 (±0.0062)	0.0211 (±0.0048)	0.0287 (±0.0037)	0.0038 (±0.0000)	0.0060 (±0.0005)	0.0330 (±0.0038)

### Runs of homozygosity

**Fig 6:**
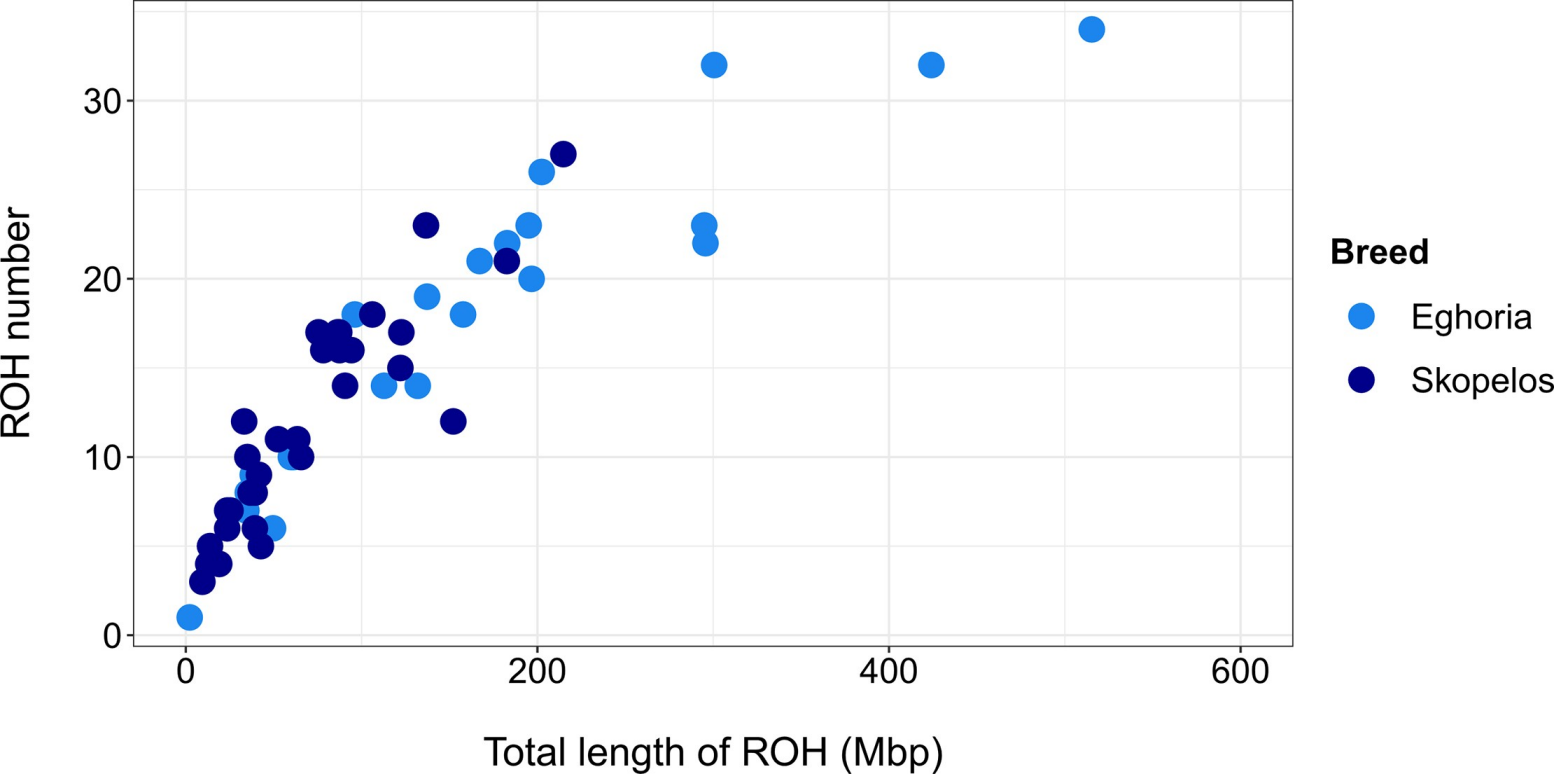
Total number of runs of homozygosity (ROHs) per animal and breed, compared to the total length of ROHs.

According to the parameters used, 765 ROHs were found in total; Eghoria had a slightly larger number of ROHs (n = 389) than Skopelos (n = 376) with an average of 12.16 and 11.75 ROHs per animal, respectively, including those that did not present any ROH (S5 Fig). The longest ROH was found in Eghoria breed (max. length in sample EG6 = 69.336 Mbp) which did not differ significantly in length compared to the longest ROH in Skopelos (max. length 60.261 Mbp for sample SK6). On average, Eghoria presented largest mean length of ROHs for the entire population (mean length = 9.488 Mbp), compared to Skopelos breed (mean length = 6.022 Mbp). Among the 765 ROHs, 51 ROHs were longer than 20 Mbp (39 for Eghoria and 12 for Skopelos), 115 ROHs were between 10 to 20 Mbp (74 for Eghoria and 41 for Skopelos) and 599 ROHs were found below the length of 10 Mb (276 for Eghoria and 323 for Skopelos breeds). Nevertheless, Eghoria demonstrated considerable differences compared to Skopelos breed concerning the total length of genome covered by ROHs (Fig 6). For instance, EG6 expresses 34 ROH segments covering over 500 Mbp of its genome, whereas, SK6 counts 27 ROH segments of 214.73 Mbp in total.

**Fig 7:**
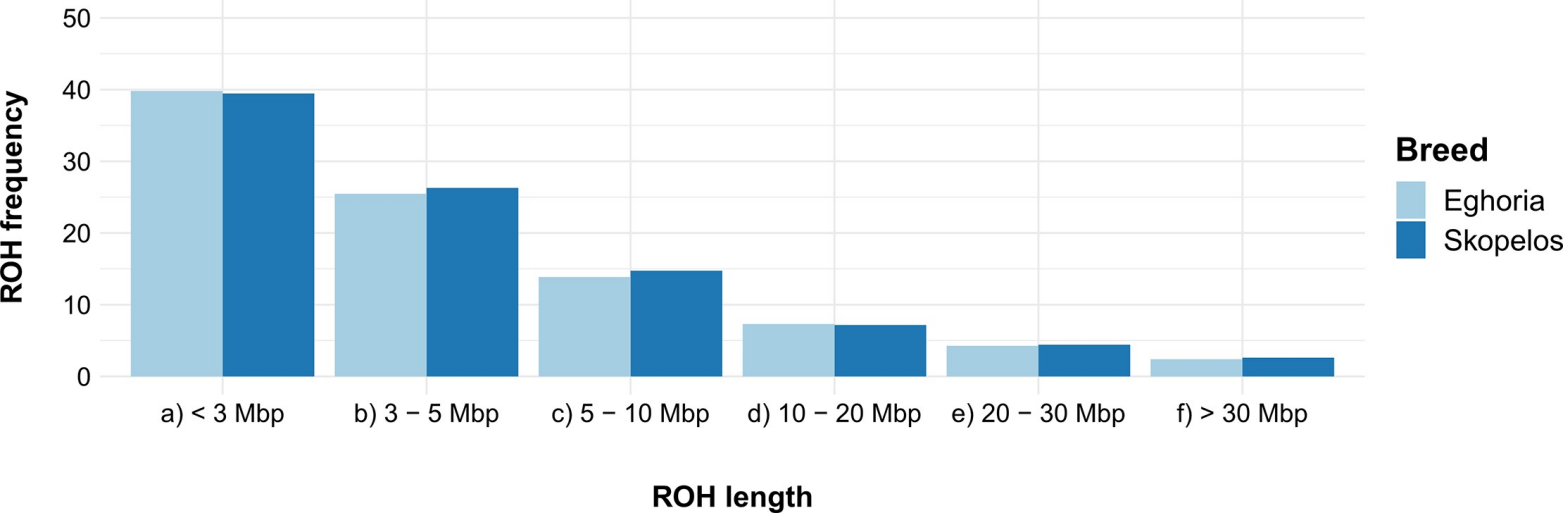
Runs of homozygosity (ROHs) frequencies per breed, classified according to their length. ROHs were classified in six length categories (<3 Mbp, 3–5 Mbp, 5–10 Mbp, 10–20 Mbp, 20–30 Mbp, >30 Mbp). Mbp: Megabase pair.

ROHs frequencies were further classified according to their size and breed. For both breeds, the frequency of short ROHs was higher compared to ROHs of greater size (Fig 7). In general, results showed that there is an inverse trend between ROH frequency and ROH length, indicating the absence of recent inbreeding among individuals within each breed. Again, no significant differences were observed among breeds.

Chromosome 6 had the largest number of ROHs (n = 48), followed by chromosome 10 (n = 47) and chromosome 1 (n = 46). Overall, the total number of ROHs per chromosome decreased with decreasing chromosome length (S6 Fig), except for chromosomes 2, 3, 4 and 5 that presented low number of ROHs and chromosome 21 that presented high number of ROHs, compared to their size. Looking at the percentage of ROHs per chromosome, the highest percentage was found in chromosome 26 (24.17%) followed by chromosome 25 (24.09%) and the lowest was found in chromosome 5 (6.06%). The average percentage of ROHs per chromosome showed an inverse relationship, with these values increasing while chromosome size decreases. Regarding SNPs located within ROHs and across autosomes, chromosome 6 had the highest frequency of SNPs in ROH segments for both breeds (S7 Fig). Although the proportion of SNPs in ROHs is below 30% for both breeds and on any chromosome, homozygous clusters can still be spotted throughout the genome.

### Linkage disequilibrium patterns

Extent of linkage disequilibrium was assessed with pairwise r^2^ in the 48,841 autosomal SNPs, for each breed separately. The pattern of LD measured by r^2^ was quite similar between breeds. The most rapid decline for both breeds was observed over the first 0.1 Mbp. Eghoria breed showed somewhat higher rates of LD decay compared to Skopelos, which displayed slightly increased levels of LD (S8 Fig). In general, LD patterns measured by r^2^ were alike between breeds according to the parameters used; mean r^2^ was 0.084 in Eghoria and 0.087 in Skopelos breed. The average inter-marker distances were about 260 kb for both breeds. Chromosomes 1 and 2 had the largest number of adjacent SNPs in LD, whereas the lowest was observed in chromosome 25; generally, the total number of adjacent SNPs in LD tend to decrease with decreasing chromosome length.

### Effective population size

**Fig 8:**
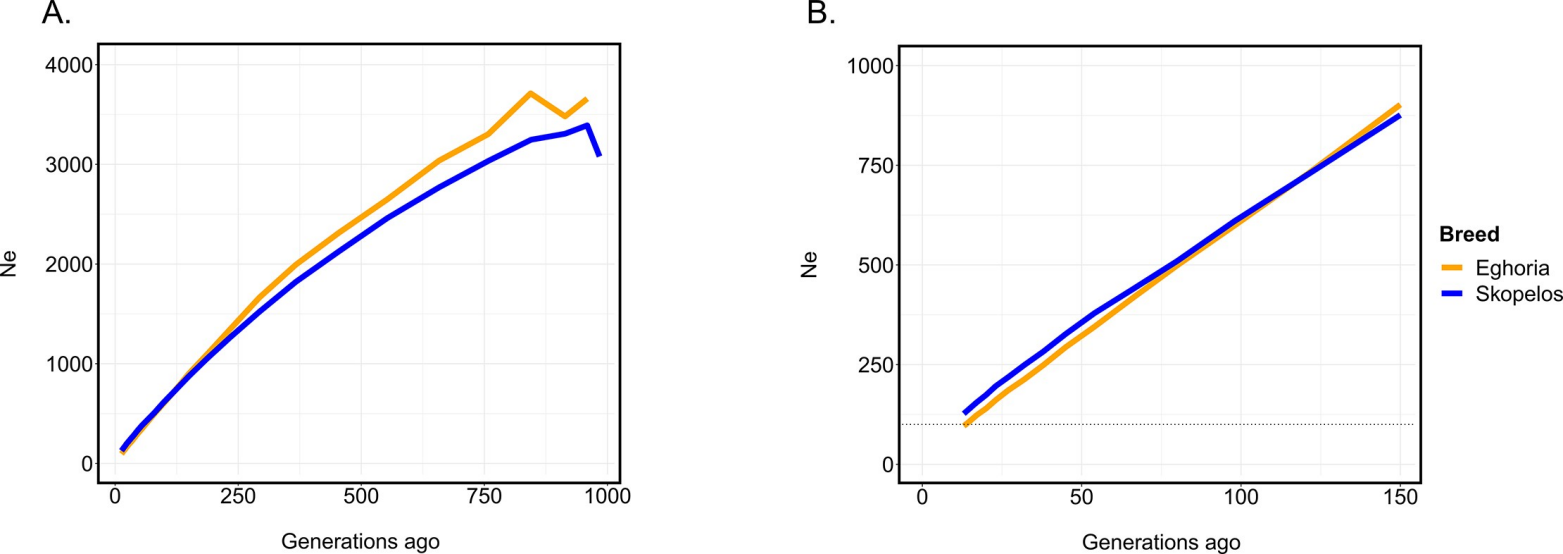
Effective population size (Ne) of Greek goat breeds. Estimation for the two goat breeds was calculated over the last A) 1,000 and B) 150 generations ago. The horizontal dotted line represents Ne = 100.

Estimates of ancestral Ne obtained for 26 time points, for the past ~1,000 generations are presented in S3 Table. Effective population size in both breeds displayed a decreasing trend over time. According to the genotyped populations, in the distant past (more than 121 generations ago) Eghoria had larger Ne compared to Skopelos breed. However, in recent past (13–98 generations ago), Eghoria presented lower Ne values compared to Skopelos. In the distant past, for the period of ~1,000 generations ago, Ne was estimated to be 3,659 and 3,391 for the Eghoria and Skopelos breed, respectively (Fig 8A). The most recent Ne values dated 13 generations ago, which equals to ~52 years ago assuming a generation interval of 4 years in goats, with the respective numbers being reduced to 96 and 127, representing a narrower genetic pool for both breeds (Fig 8B).

### Discriminatory SNPs between breeds

**Fig 9:**
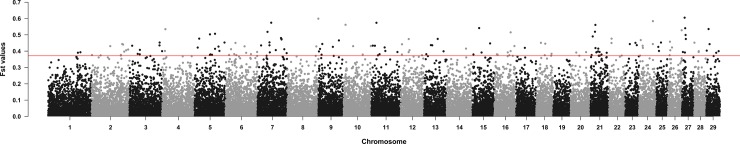
Manhattan plot of the Fst values for each single nucleotide polymorphism (SNP). Chromosomes are alternately colored in black and grey. Red line corresponds to the 0.995 of the percentile distribution (Fst = 0.372).

Genome-wide association analysis was applied to the dataset of 48,841 SNPs for all animals, to identify markers for breed discrimination and origin assignment. To that end, three approaches were used. Using W&C’s Fst approach, 151 SNPs were detected at a threshold of 0.372 (equal to the 0.995 of the percentile distribution) (Fig 9). Discriminatory SNPs were located on 28 autosomes; no SNP was detected above the selected threshold for chromosome 19. The highest number of significant SNPs was observed for chromosomes 7, 21 and 5, counting 12, 12 and 11 SNPs, respectively. Using the W&C’s algorithm negative values were obtained for 18,724 SNPs, indicating that there is more variation within than between the studied breeds.

The 151 discriminatory SNPs are located within or ±100kb of 381 genes. SNPs with the highest Fst value were snp30300-scaffold333-3618181, snp17056-scaffold178-6924 and snp7659-scaffold1277-264730, located on chromosomes 27, 8 and 4, respectively. The 151 identified SNPs, alongside with the detected genes in nearby regions are summarized in S4 Table. Interestingly, among the identified genes, a cluster of tRNA genes was found at a higher frequency, and in particular, *TRNAC-GCA* (transfer RNA cysteine (anticodon GCA)) was encountered as the nearest gene of many discriminatory SNPs.

Identification of SNPs for breed assignment using the TRES_all approach and the algorithms implemented in TRES software led to 155 common SNPs among the three methodologies (similarity percentage 77.5%) (S5 Table). Discriminatory SNPs were located on 28 autosomes; similar to the Fst approach, no discriminatory SNP was detected on chromosome 19. Chromosomes 1 and 21 had the largest number of discriminatory SNPs (N = 13 SNPs for each chromosome). In total, 389 unique genes were identified within ±100kb of the identified SNPs; among them, genes that were located in the nearby regions of the SNPs with the highest Fst value, were also present. Algorithms implemented in TRES also indicated that *TRNAC-GCA* was the most frequent neighboring gene of the 155 discriminatory SNPs.

The independent identification of SNPs in the training population (TRES_tt methodology) resulted in 157 SNPs that corresponded to a similarity percentage of 78.5% among the three methodologies implemented in TRES software. SNPs were located in all autosomes; chromosomes 7, 6 and 5 presented the highest number of discriminatory SNPs with 19, 13 and 10 SNPs, respectively. Four-hundred six genes were identified in the nearby regions (within ±100kb) of the 157 SNPs (S6 Table). Evaluation of the 157 SNPs in the test population using GeneClass2 correctly assigned all individuals in the test population.

Common SNPs among the three methodologies were further evaluated in GeneClass2 software. An overlap of discriminatory SNPs per methodology was observed (S9 Fig). In particular, 95 SNPs (42.6%) were common among the three methods (presented in bold in S4–S6 Tables). It is noteworthy that W&C’s Fst and TRES_all methodologies shared 135 discriminatory SNPs (78.9%). Evaluation of the 95 SNPs using different genetic assignment methods correctly assigned all individuals according their origin (S7 Table). Despite the fact that each individual was correctly assigned to the most likely source population at a strict pre-specified confidence level, assignment probabilities (A.P.) for some animals were low. For the Eghoria breed, these animals originated from farm F4, whereas Skopelos animals with low A.P. originated from farms F3, F5 and F6. Generally, the more admixed an individual was found based on previous analyses (e.g. admixture), the lower A.P. values it presented. Yet, some cases were observed in which evaluation of individuals using the GeneClass2 software did not agree with admixture analysis. For example, samples SK6, SK10 and SK32 demonstrate identical profile at K = 3 in admixture analysis, whereas the A.P. value for Skopelos breed for SK10 is lower compared to SK6 and SK32 animals.

Eighty-four out of the 95 common SNPs were found to be in ROH segments either for Eghoria or Skopelos breeds. The 95 common SNPs were located in or within ±100kb from 237 genes. Among them, some well-characterized genes were included such as *BMP2* (Bone morphogenetic protein 2), *PDGFRB* (b-Type platelet-derived growth factor receptor) and *ZAR1* (Zygote arrest 1). The 95 common SNPs were not evenly distributed across autosomes; chromosome 7 had the largest number of identified discriminatory SNPs (N = 12), followed by chromosome 21 (N = 8) and 5 (N = 8).

## Discussion

### Genetic diversity

In this study the population structure, genetic diversity and inbreeding levels were examined in the two recognized Greek goat breeds, Eghoria and Skopelos, using Illumina’s Goat SNP50 BeadChip. Overall, the populations studied here possess high levels of genetic diversity according to the indices used. The use of Goat SNP50 BeadChip identified large numbers of polymorphic loci in both breeds, despite the fact that its design and initial validation was based on other breeds for different breeding purposes (Alpine, Angora, Boer, Creole, Jinlan, Kacang, Saanen, Savanna, Yunling). Calculated heterozygosity values of Greek breeds (mean Ho = 0.394, mean He = 0.399) were in agreement with most published studies employing Goat SNP50 BeadChip. Compared to the average heterozygosity values of 43 European breeds reported by Colli et al., (mean Ho = 0.369, mean He = 0.378) [14], Greek breeds presented slightly higher heterozygosity levels. This was also observed focusing only on breeds reared in the Mediterranean basin such as Spanish [55] or Italian goat breeds [56]. Our heterozygosity results were similar with four goat breeds from Sudan (mean He = 0.400) [17], the Spanish Bermeya breed (mean He = 0,399) alongside with Boer (mean He = 0,399) and Toggenburg (mean He = 0,399) populations reared in Eastern Africa (Uganda, Kenya, Tanzania) [14]. However, results with such small differences are not significant, especially considering that in a cosmopolitan breed such as Toggenburg, different He values have been reported in purebred nuclei, ranging from 0.336 [21] to 0.431 [57] using the same genotyping beadchip. These variations could be strongly dependent on sampling locations. Inconsistencies in heterozygosity results were also observed in Angora purebreds reared in three different continents in which the influence of genetic and geographical isolation as well as different selection patterns showed some discrepancies between the studied populations, mostly indicated by their pairwise F_ST_ values [58]. In our study, the low value of the F_ST_ index (F_ST_ = 0.04362) indicates that Greek goat breeds are genetically related based on the SNP dataset used; since values of this magnitude (i.e. close to 0) indicate low differentiation between breeds or that historical gene flow exists between breeds. This result is not in agreement with a previous study on the two autochthonous Greek breeds which generated an F_ST_ value of 0.10000; however, in their study only a small number of SNPs (n = 26) was analyzed [5]. To our knowledge, Greek goat breeds have never been genotyped with a genome-wide DNA array; as such, we were not able to directly compare and confirm our results with other datasets.

### Population structure

Analysis of Greek goat breeds using 45 breeds reared worldwide revealed that Eghoria and Skopelos cluster together with European breeds, away from African and Asian populations. The clustering of this continental subdivision, and in particular the grouping of Greek breeds with Italian (JON, RME, GAR, ASP, ARG, MLT in Figs 1 and 2) Carpatian and Spanish breeds (CRP and MUG in Figs 1 and 2, respectively) provides insights into the migration waves from the domestication center to the formation of the present European gene pool. Our findings support the hypothesis reported by Colli et al. [14], which suggested that goats from Central and East Mediterranean regions were influenced by West Africa and Southwest Asia at the same time, thus confirming the role of the Mediterranean basin as a crossroad of post-domestication [14]. However, the obtained bootstrap values from gene-flow analysis for Eghoria and Skopelos breeds in our dataset suggest that another resampling could lead to different reconstruction of their nodes, thus, different population splits and gene flow events.

It is important to highlight that although the advent of genome-wide technologies have created cosmopolitan breeds that are far more productive than the local ones, admixture among Greek and cosmopolitan breeds remains at low levels, despite the fact that in Greece many breeders tend to neglect indigenous, less productive breeds.

Concerning the Greek population structure, our study reveals a geographical pattern, grouping individuals according to the sampling locations. All animals from the northwestern Greece formed a tight cluster without overlaps with clusters of other geographical origin. The tight and distinct cluster observed in individuals from farm 1 reflects the limited gene flow among goat populations in that region due to the presence of geographical barriers (mountains) (S10 Fig). This was also observed in Skopelos breed which is expected since insularization usually involves increased levels of homozygosity as a result of management practices, history and demography [13]. The genetic distance observed between the two Eghoria populations (farms 1 and 4) reflects the large genetic variation that this breed expresses which is mainly related to the colonization of Eghoria purebreds in different geographic areas. The small sample size analyzed in the present study, alongside with the limited number of farm locations used for sampling could influence the results reported here concerning the genetic diversity of this breed. Hence, the observed geographical pattern raises questions regarding the population structure of all the recognized goat types in Greece, especially for the indigenous Eghoria breed. Thus, it would be interesting to explore population structure among populations located in geographically isolated regions and in inaccessible areas or where transhumance is still active.

Population structure analyses revealed that the most admixed population inhabits central Macedonia (northern Greece), which is a region featuring numerous goat farms with different production systems. ADMIXTURE and PCA analyses in farm 4 indicate that admixture between purebreds existed in the past, with exchanges of genetic material between breeds, despite the fact that their phenotypes pointed otherwise. The high levels of admixture in farm 4 in our study are possibly due to an unsupervised and random mating system between animals, which is usual in goat faming in Greece. The grouping of individuals according to the sampling locations evidenced in our results has previously been reported for several goat breeds, where gene flow was studied in their transit from Middle East to the Iberian Peninsula [59, 60].

### Patterns of homozygosity

Runs of homozygosity in autosomal SNPs were determined for all samples in each breed, to assess whether regions in the genome are present, that are potentially under positive selection, therefore indicators of selective sweeps in the studied populations [61, 62], or to provide insights on how demography and the recombination landscape have evolved over time for the two breeds [63, 64]. The high levels of genetic diversity in the two breeds, indicated by heterozygosity levels (Ho, He) and Wright’s F statistics, were also confirmed by analyzing the degree of inbreeding through indices such as F_IS_ and F_ROH_. The degree of inbreeding for the two breeds was quite similar in all inbreeding coefficients measured. Since F_IS_ values did not deviate significantly from zero in both breeds, we cannot not claim that Greek breeds are a result of genetic subdivision (Wahlund effect). The same pattern in F_IS_ values is observed in many cosmopolitan goat breeds reared worldwide such as Angora, Alpine, Saanen and Toggenburg [57, 58], as well as in many autochthonous breeds in Italy [56], Sudan [17] and South Africa [65].

Since there is no consensus or standardized criteria to define a ROH within a species, we identified ROH segments in the goat genome, by applying the same parameters used by the AdaptMap project [11, 13]; therefore, our results could be comparable with other breeds reared worldwide. In the present study, inbreeding was also calculated through ROHs, since many studies have shown that F_ROH_ seems to be a more powerful approach for detecting inbreeding than any other alternative method, due to the advantage of not being influenced by estimates of allele frequency or incomplete pedigree data [66–68]. Estimates of pedigree-based inbreeding could not be derived in the present study due to lack of pedigree data in the studied animals. The average fraction of the genome that contains ROH for Eghoria was higher compared to Skopelos breed. Skopelos displayed low F_ROH_ value, similar to breeds from East Africa (Galla, Karamonja, Sonjo, Sebei, Woyito Guji, Gumez), Turkey (Kil, Kilis) and some European breeds from Italy (Argentata), Spain (Malaguena, Bermeya) and Romania (Carpatian) [11, 13]. The increased F_ROH_ value for Eghoria compared to Skopelos breed was equivalent to autochthonous goat breeds from Europe (Valpassiria, Garganica, Ciociara Grigia, Provencale, Alpine) and Africa (Saidi, Burundi, Matebele, Red Sokoto, Guera, Nicastrese, Pare White, Keffa) [11, 13].

According to our results, Greek breeds presented quite smaller number of ROHs per individual but a higher average ROH length compared to various breeds reared worldwide [21] or local breeds [55]. The opposite was true for Swiss goat breeds which presented a quite higher average ROH length (max ROH length = 103.745 Mb) [69]. Interestingly, Eghoria exhibited greater genetic diversity based on the other inbreeding indices but had higher F_ROH_ compared to Skopelos breed. This has been also reported for the Rangeland breed reported by Brito et al. [21] and can be justified by recent selection or inbreeding. Another possible explanation of the increased homozygosity in Eghoria breed could be justified by the consecutive expansions of Eghoria populations over the Greek mainland, thus several founder effects and geographical isolation could be responsible for the increased homozygosity levels. Additionally, many useful SNPs might be omitted from the medium density array used, which could corrupt the ROH segments and lead to different results. True extent of homozygosity can be underestimated due to not clearly defined SNPs, e.g. hemizygous deletions, or due to the fact that sometimes SNPs are not LD-pruned before the data can be used for ROH analysis [70, 71]. Probably, a denser SNP panel, would normalize such inconsistencies and improve prediction accuracies, although it has been documented that increased marker density may improve resolution, but can also decrease power and add noise to the analyses by the use of non-informative SNPs [72].

ROH analysis revealed that many of the detected SNPs within ROHs mapped to genes with well-characterized functions. For both breeds, chromosome 6 demonstrated large homozygous regions, with SNPs within these ROHs mapping to genes affecting milk production traits such as the casein gene cluster containing *CSN1S1* (alpha-S1-casein), *CSN1S2* (alpha-S2-casein), *CSN2* (beta casein) and *CSN3* (kappa casein) [73] or the *ABCG2* (*ATP binding cassette subfamily G member 2*) gene [74]. Moreover, ROH segments were also found to map to the *BMPR1B* (bone morphogenetic protein receptor) gene, which is known as a major gene for prolificacy in sheep [75], however its role in goats concerning prolificacy is still unclear and remains to be explored [76–79]. ROH analysis also revealed regions on chromosome 14, covered by multiple ROH segments for the Eghoria breed (15.2–81.5 Mb); within this region *DGAT* (Diacylglycerol O-Acyltransferase 1) gene is located, which has recently been found to affect milk fat content in dairy goats [80].

Linkage disequilibrium analysis through r^2^ confirmed the results obtained from ROH analysis, although differences between breeds were quite modest. In particular, the slightly lower LD decay in Eghoria corroborated increased levels of homozygosity compared to Skopelos breed. Mean r^2^ values were lower than other goat breeds [57, 81, 82] but, in general, the pattern of LD decay was a typical curve that is usually observed in livestock species [83–85]. However, setting a threshold for r^2^ of 0.3 to ensure successful whole genome association studies [86] and considering the rapid LD decay over short distances [37, 87], we highlight once more the need for a denser SNP panel. Consistent with the results obtained from ROH analysis, the short range of LD proves that Greek breeds differ from the intensively selected ones such as Cashmere, Toggenburg and Boer [81] in which LD decay, as well as their respective effective population sizes, point otherwise.

Despite the differences in their morphological and productive characteristics, Greek breeds seem to share a similar genetic background. Due to breeding practices (intense selection through participation in conservation programs), history and demography of Skopelos breed, we would expect increased levels of homozygosity compared to Eghoria breed. Yet, our results indicated that the two breeds demonstrate similar levels of genetic diversity. Furthermore, accepting 50–100 as a critical effective population size range for long-term viability [88], our results reveal that both Greek goat breeds are of considerable sizes and are not being threatened.

### Breed assignment

Discrimination of goat breeds has been studied in the past using microsatellite markers [89]; however, the power of analysis increases when genotyping microarrays are used instead, since associations rely on a much higher number of markers and for this reason microsatellites have now been replaced in the study of breed identification [16, 90, 91]. In our study, by using the Goat SNP50 BeadChip, admixed animals were traced, although their available recorded pedigree data pointed otherwise. Hence, since pedigree data are very limited in Greek indigenous goats, we propose that an alternative way is possible to better assign individuals to their origin, mostly including genomic data. In the present study, we applied three different methods to select a reduced and representative number of markers from Goat SNP50 BeadChip to efficiently discriminate Greek goat breeds.

Population structure and breed assignment using W&C’s Fst and TRES approaches have been evaluated in goats [12, 21, 69, 92] and avian species [93, 94]. A panel of informative SNPs for parentage assignment in goat breeds reared worldwide have recently been published by the AdaptMap initiative [16], however, no SNP was common with our dataset. In our dataset, algorithms implemented in W&C’s Fst and TRES methodologies resulted in a high percentage of common discriminatory SNPs. However, further evaluation of the discriminatory SNPs is required, including phenotypic data and subsequent validation in populations from different areas before these SNPs can be efficiently used in animal breeding programs, probably through the design of a customized Greek goat SNP panel. Additional validation of our results with larger population sizes analyzed would be desirable, since the small sample size analyzed in this study could influence the obtained results. With the recent update of the goat genome assembly [9] it is expected that non annotated SNPs will be assigned to genomic locations and associated with biological functions, and the need highlighted by many authors for a denser genotyping microarray in goats will be fulfilled, making studies on population genetics even more accurate.

An important problem highlighted from the present study is the incorrect classification of purebreds despite their recorded pedigree and morphological characteristics, emphasizing the need for a more effective way to identify and record individuals. Preservation of indigenous purebreds is crucial since the irreversible loss of alleles could lead to decreased adaptability and survival rates especially taking into consideration the threat from the upcoming climatic change [95]. Since goats are among the livestock species that will be directly affected by these changes, well adapted individuals should be exploited to breed the resilient genotypes.

Within ±100kb of the 95 common discriminatory SNPs many candidate genes were found. All the identified genes surrounding the 95 SNPs (237 genes), were extracted and used as a dataset to search against the KEGG database for metabolic pathways relevant to important productive traits (data not shown). Based on this analysis, only general/basic pathways, essential for life maintenance and organism homeostasis were detected e.g. propanoate metabolism, cell cycle, amino sugar and nucleotide sugar metabolism. Among the identified loci, *BMP2* is a well characterized gene, that has been associated in sheep with body size development [92] and tail-type formation [96]. In goats, *BMP2* has been identified as a putative gene involved in the formation and adaptation to the alpine environment of Swiss goat populations [69] and as a regulator of hair follicle development in Cashmere goats [97]. Another gene that was found within the selected window of the discriminatory SNPs was *MYOZ1* (Myozenin 1). This gene has been associated with different lipid traits in cattle [98] and with fetal weight in sheep [99]. In goats, however, its role remains to be explored, although members of the same family (*MYOZ2* and *MYOZ3*) have been studied against their role in meat quality [100]. Moreover, our results indicate that *ZAR1* gene is identified in the nearby regions of discriminatory SNPs, in all methodologies applied. *ZAR1* is a gene well known for its role on embryonic development and fertility control in mammals [101]. Yet, in goats this gene has not been intensively studied, though it was found to be located in a QTL under selection in Saanen goats [21]. *TRNAC-GCA* is a gene reported by many authors that is located around significant or discriminatory SNPs. Specifically, QTLs enclosing this gene have high effect in carcass weight [102] and in mineral (Cr, Fe) concentration in muscles [103] in Nelore cattle. In addition, *TRNAC-GCA* has been related to affect residual feed intake in Angus breed [104] and sperm quality in Holstein-Friesian bulls [105]. In goats, *TRNAC-GCA* has been frequently encountered in genomic regions showing evidence of positive selection for breeds of different purposes [21].

## Conclusions

In this study, genetic diversity and population structure of autochthonous Greek goat breeds was assessed for the first time using the Goat SNP50 BeadChip. Our results provide a comprehensive characterization of goat genetic diversity, inbreeding levels and population structure in Greece, using ~60K SNPs. Our findings revealed that the two indigenous goat breeds maintain high levels of genetic diversity which can be further exploited in the design and implementation of breeding schemes. Eghoria presented slightly increased inbreeding levels, nevertheless, our results show that the two breeds are closely related. Moreover, first insights of Greek goat breed traceability are presented, hence, our data could be used for breed discrimination and protection of the authenticity of traditional local products. According to our results, the high levels of genetic diversity in Greek goat breeds could serve as a valuable genetic reservoir to be exploited in national breeding schemes.

## Supporting information

S1 Fig Geographical map indicating the location of goat farms. Sampling for the Eghoria breed was conducted from farm 1 (F1, number of animals (N) = 15) located in Tsepelovo and farm 4 (F4, N = 17) located in Thessaloniki. Sampling for the Skopelos breed was conducted from farm 2 (F2, N = 10), farm 3 (F3, N = 10) and farm 6 (F6, N = 9) located in Skopelos, farm 4 (F4, N = 8) and farm 5 (F5, N = 3) located in Alonnisos island.
(TIFF)

S2 Fig Fraction of variance explained per migration event (m) using the Treemix software. Each migration event was analyzed in triplicates.
(TIFF)

S3 Fig Plot of residuals when ten migration edges were fit. (TIFF)

S4 Fig Admixture analysis per farm at K = 2 and 3. Each farm is presented by a vertical bar. Different colors indicate different clustering groups. F1: farm 1; F2: farm 2; F3: farm 3; F4: farm 4; F5: farm 5; F6: farm 6.
(TIFF)

S5 Fig Total number and length (Mbp) of runs of homozygosity (ROHs) per animal. Different colors indicate the two breeds (orange for Eghoria, blue for Skopelos).
(TIFF)

S6 Fig Distribution of Runs of Homozygosity (ROHs) on chromosomes. Each bar represents the total number of ROHs distributed per chromosome (Number of ROH). The red line shows the average percentage of ROHs for each chromosome.
(TIFF)

S7 Fig Manhattan plot of the proportion of times (%) SNPs are located within ROHs per breed, plotted against SNP position in autosomes. Autosomes are alternately colored in red and blue. SNPs: Single nucleotide polymorphisms, ROHs: Runs of homozygosity.
(TIFF)

S8 Fig Linkage disequilibrium (LD) decay over autosomal chromosomes. (TIFF)

S9 Fig Venn diagram presenting the common and unique SNPs (Single Nucleotide Polymorphisms) per methodology. (TIFF)

S10 Fig Treemix analysis with 1 migration event for the Greek populations. (TIFF)

S1 Table Information on farms, sampling locations and number of animals analyzed per breed. (DOCX)

S2 Table Quality control of caprine Single Nucleotide Polymorphisms (SNPs). (DOCX)

S3 Table Estimates of ancestral effective population size (Ne) over past generations. (DOCX)

S4 Table List of the 151 SNPs identified using the Weir and Cockerham’s algorithm. Genes located within ±100kb of the identified SNP or nearby genes of the identified SNPs are presented in italics. Texts in bold indicate the 95 common SNPs identified among the three methods. CHR: chromosome, kb: kilo base pair; SNP: Single nucleotide polymorphism.
(DOCX)

S5 Table List of the 155 single nucleotide polymorphisms (SNPs) identified using all animals (TRES_all method). Genes located within ±100kb of the identified SNP or nearby genes of the identified SNPs are presented in italics. Texts in bold indicate the 95 common SNPs identified among the three methods. CHR: chromosome, kb: kilo base pair; SNP: Single nucleotide polymorphism.
(DOCX)

S6 Table List of the 157 single nucleotide polymorphisms (SNPs) identified by splitting the dataset into training and test populations (TRES_tt method). Genes located within ±100kb of the identified SNP or nearby genes of the identified SNPs are presented in italics. Texts in bold indicate the 95 common SNPs identified among the three methods. CHR: chromosome, kb: kilo base pair; SNP: Single nucleotide polymorphism.
(DOCX)

S7 Table Assignment probabilities for each individual using 95 SNPs, calculated with the GeneClass2 software. A.P.: Assignment Probability, SNPs: Single nucleotide polymorphisms.
(DOCX)
