# Supplementary figures and images for: Analysis of genome-wide DNA arrays reveals the genomic population structure and diversity in autochthonous Greek goat breeds
Source: PLoS One. 2019 Dec 12;14(12):e0226179. doi: 10.1371/journal.pone.0226179 (PMC6907847; doi:10.1371/journal.pone.0226179)

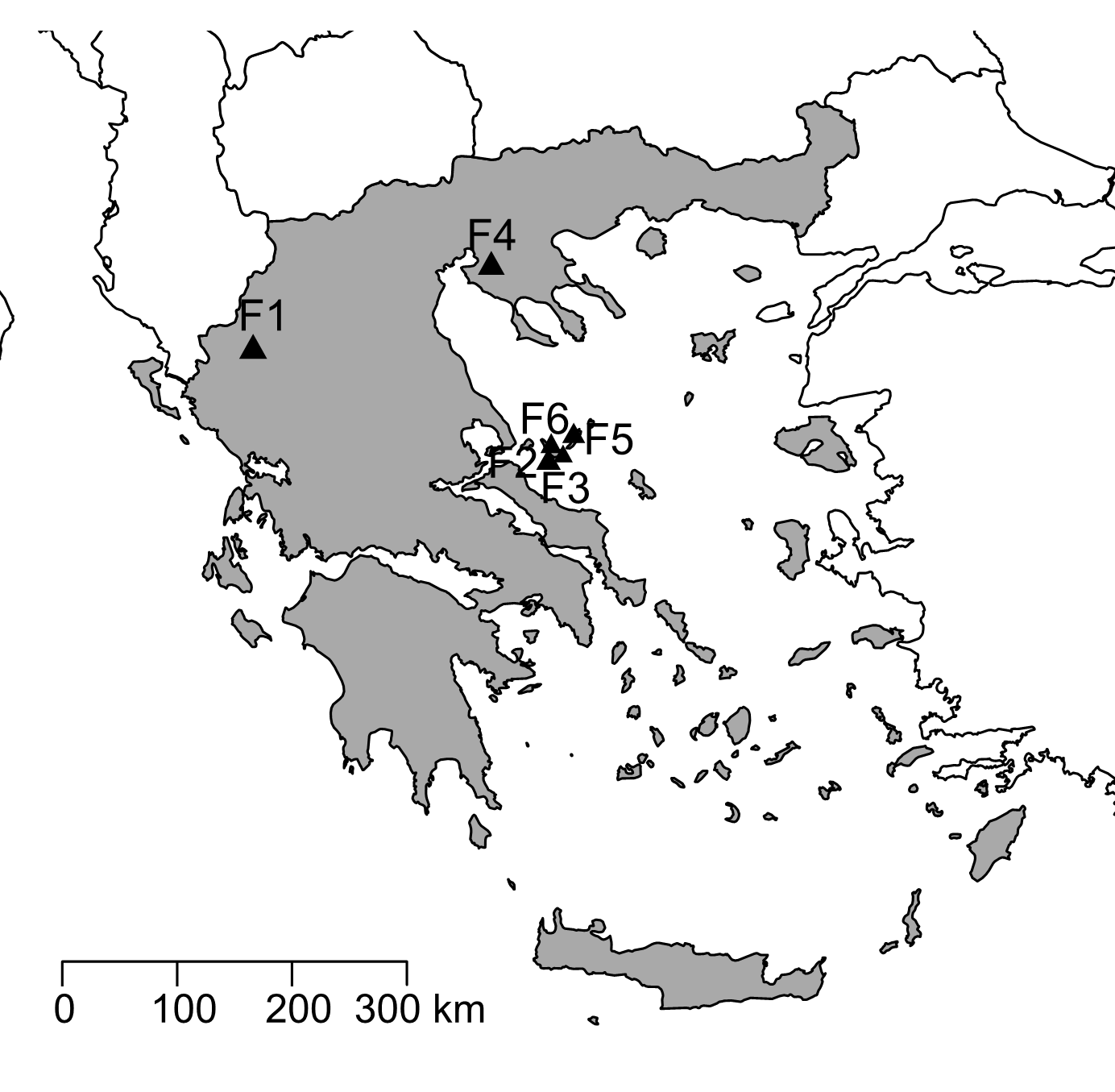

Supplement: S1 Fig — Sampling for the Eghoria breed was conducted from farm 1 (F1, number of animals (N) = 15) located in Tsepelovo and farm 4 (F4, N = 17) located in Thessaloniki. Sampling for the Skopelos breed was conducted from farm 2 (F2, N = 10), farm 3 (F3, N = 10) and farm 6 (F6, N = 9) located in Skopelos, farm 4 (F4, N = 8) and farm 5 (F5, N = 3) located in Alonnisos island. (TIFF) [file pone.0226179.s001.tiff]

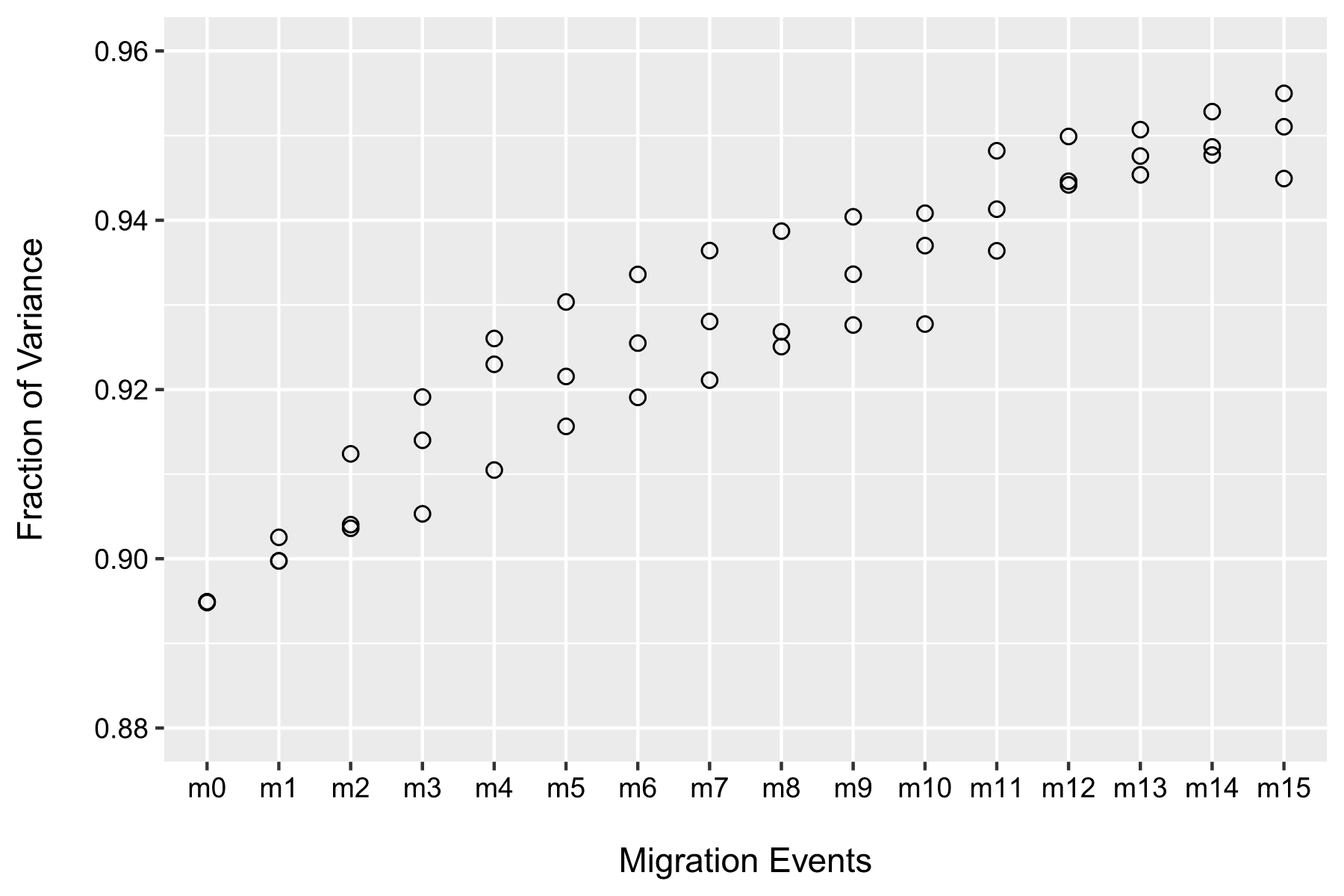

Supplement: S2 Fig — Each migration event was analyzed in triplicates. (TIFF) [file pone.0226179.s002.tiff]

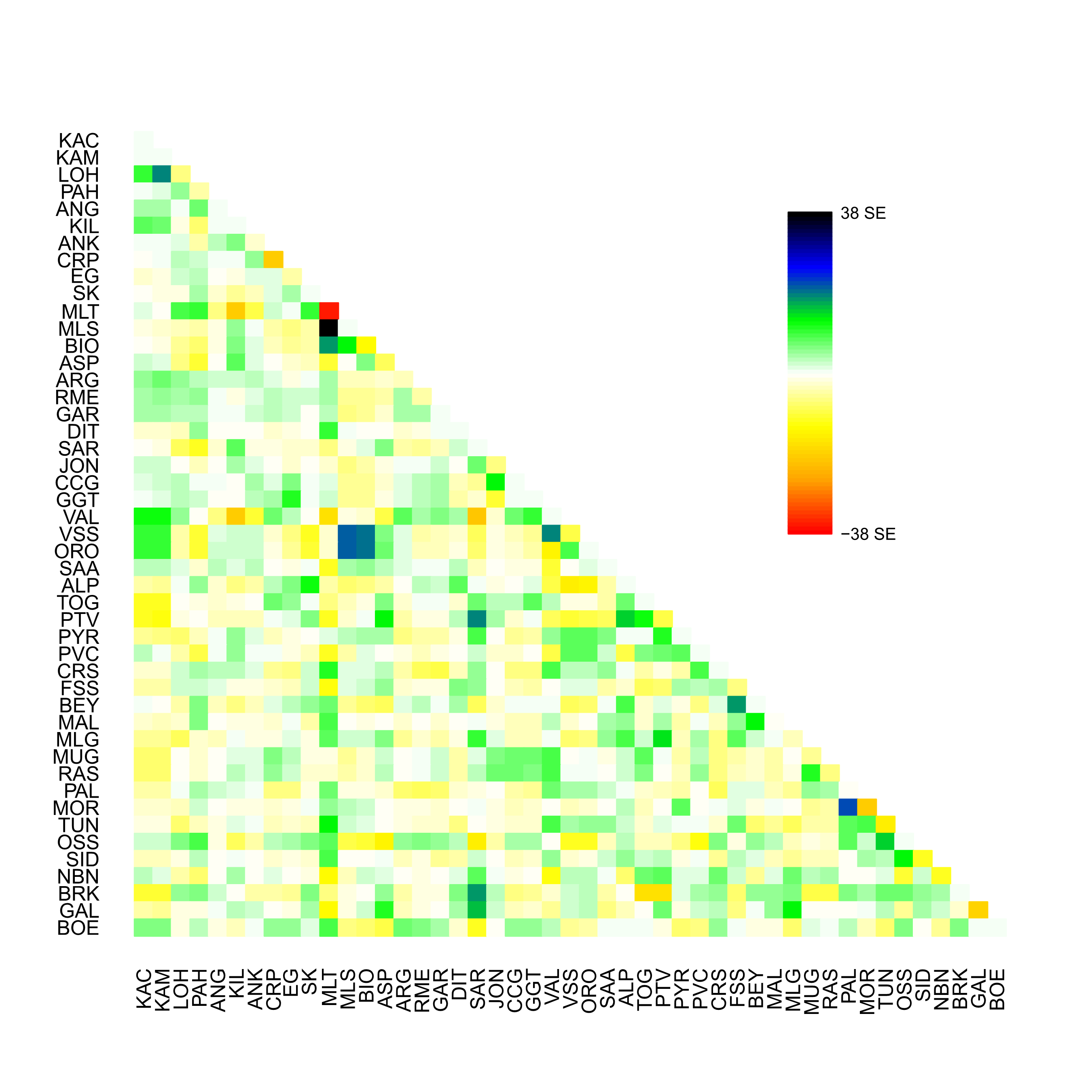

Supplement: S3 Fig — (TIFF) [file pone.0226179.s003.tiff]

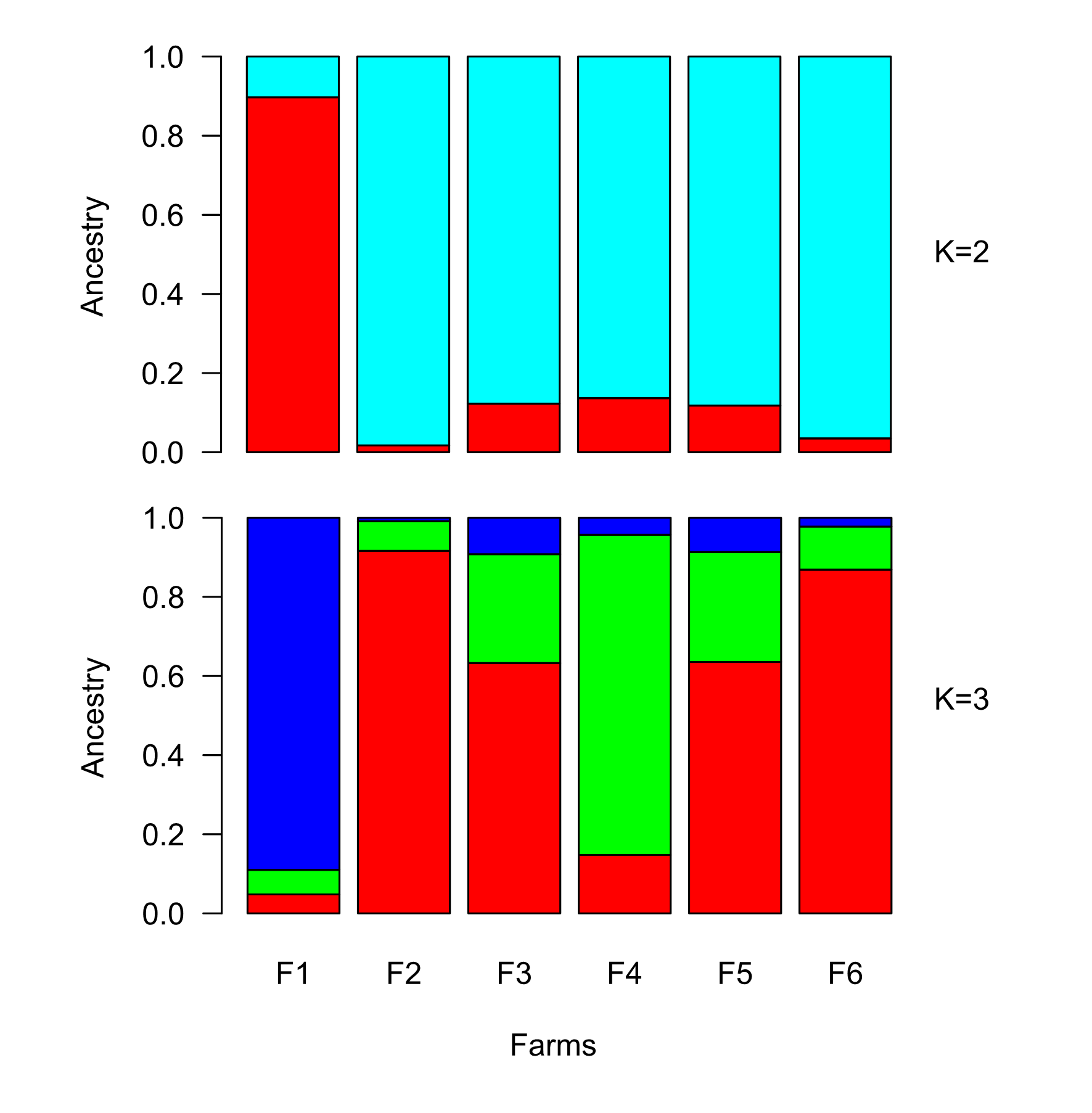

Supplement: S4 Fig — Each farm is presented by a vertical bar. Different colors indicate different clustering groups. F1: farm 1; F2: farm 2; F3: farm 3; F4: farm 4; F5: farm 5; F6: farm 6. (TIFF) [file pone.0226179.s004.tiff]

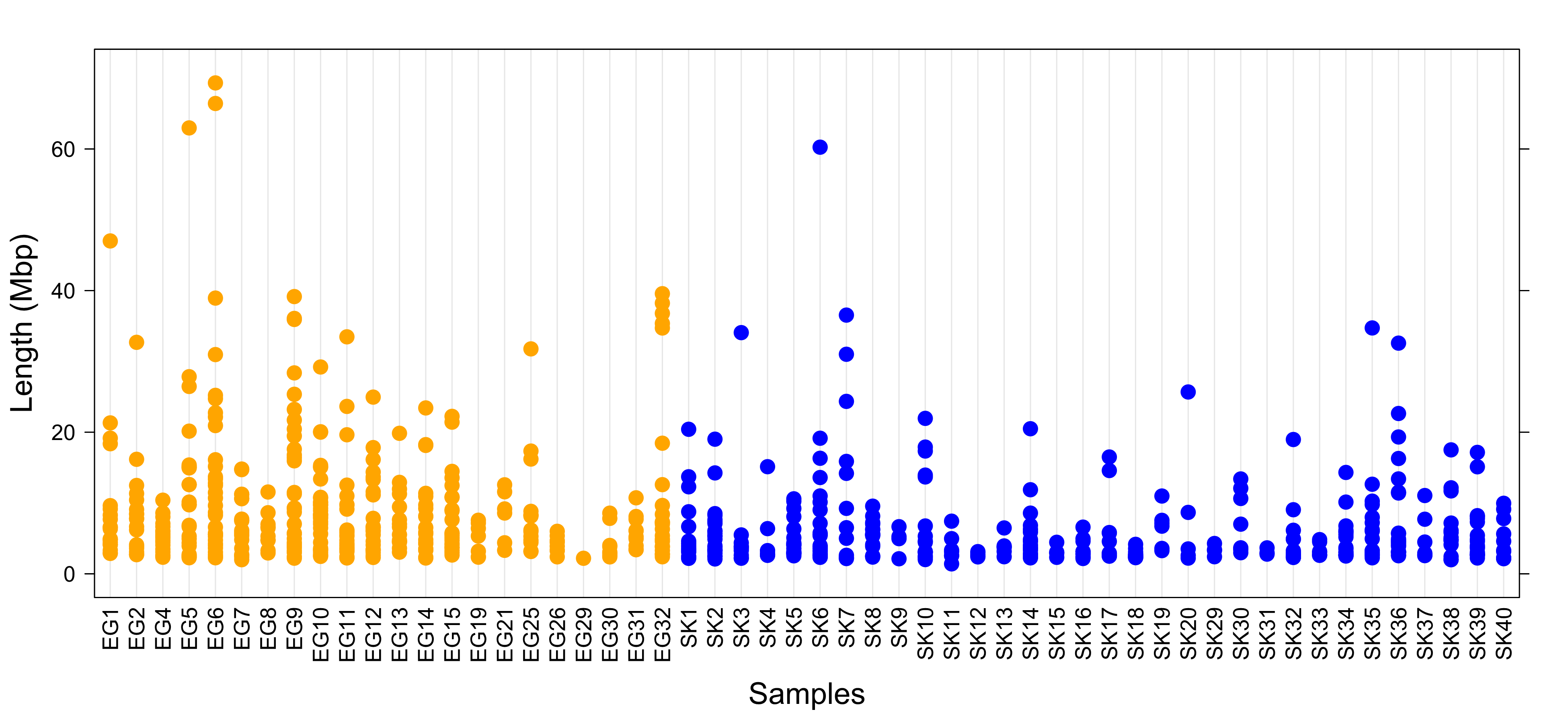

Supplement: S5 Fig — Different colors indicate the two breeds (orange for Eghoria, blue for Skopelos). (TIFF) [file pone.0226179.s005.tiff]

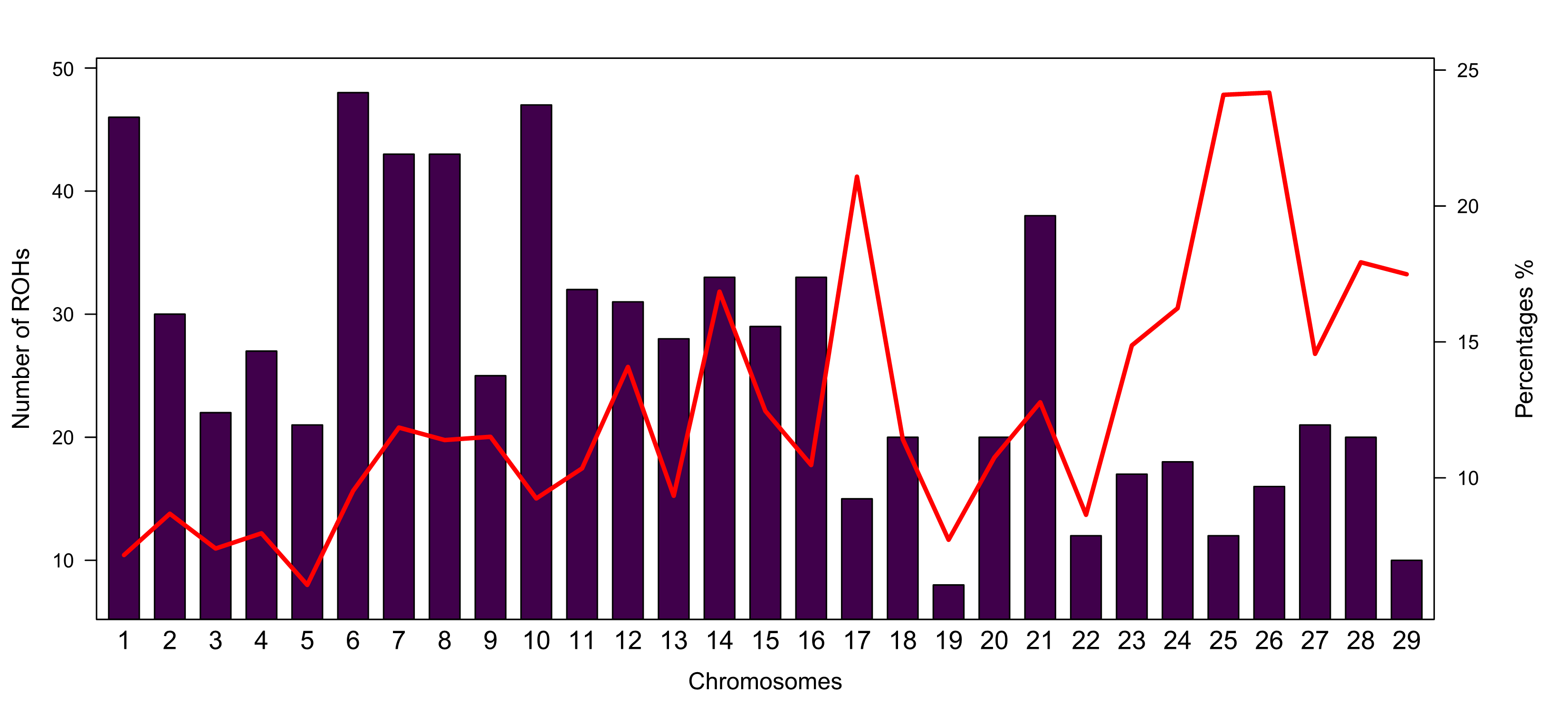

Supplement: S6 Fig — Each bar represents the total number of ROHs distributed per chromosome (Number of ROH). The red line shows the average percentage of ROHs for each chromosome. (TIFF) [file pone.0226179.s006.tiff]

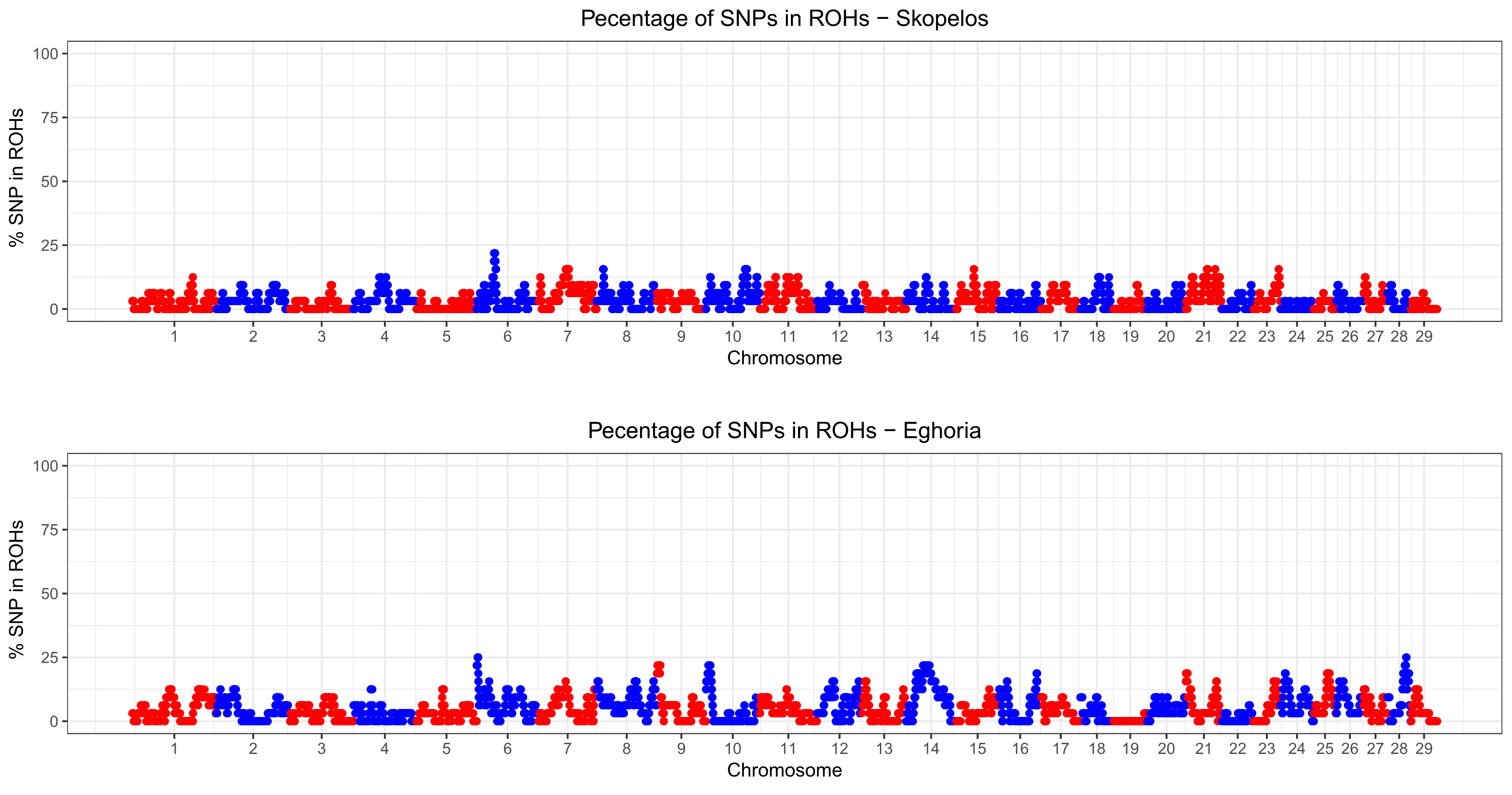

Supplement: S7 Fig — Autosomes are alternately colored in red and blue. SNPs: Single nucleotide polymorphisms, ROHs: Runs of homozygosity. (TIFF) [file pone.0226179.s007.tiff]

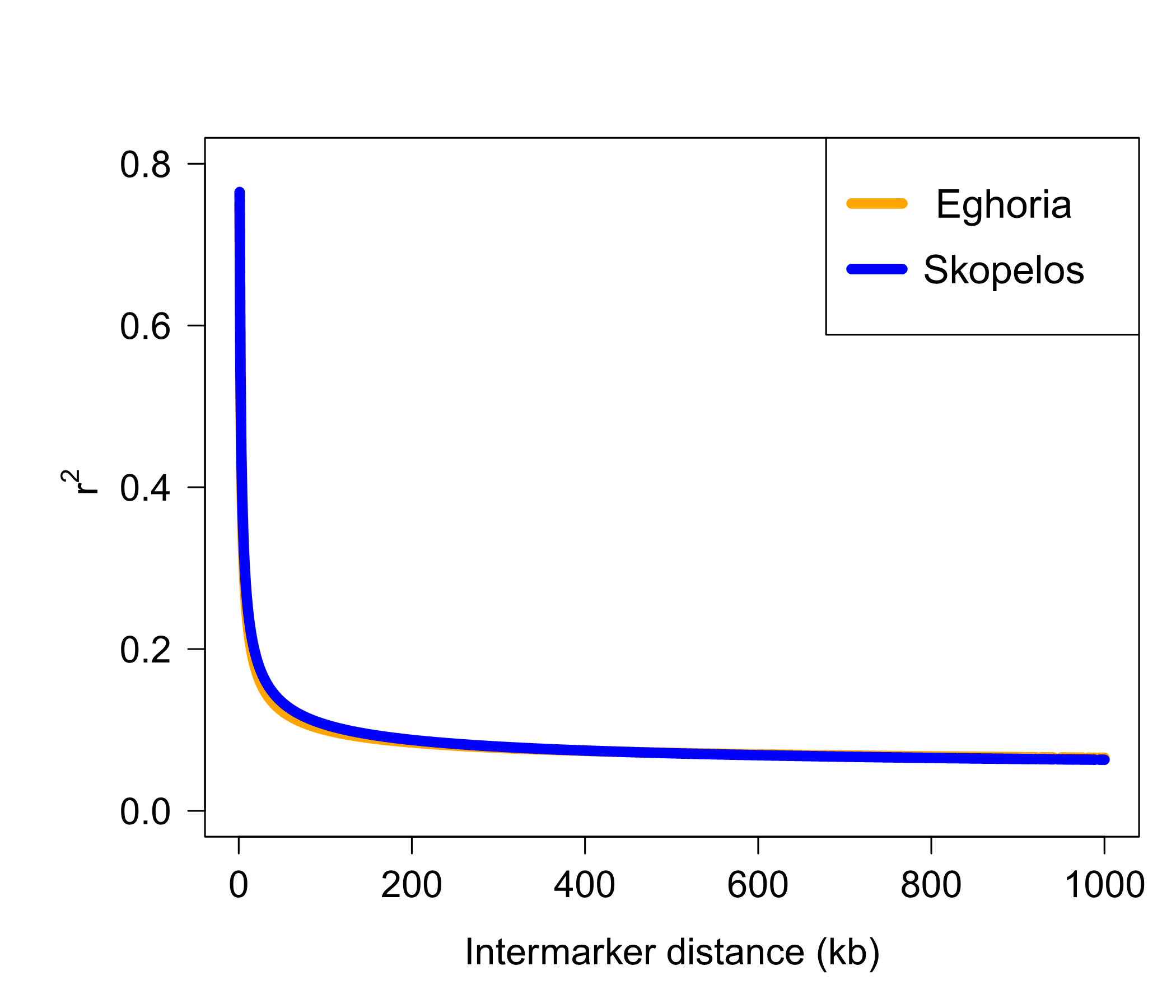

Supplement: S8 Fig — (TIFF) [file pone.0226179.s008.tiff]

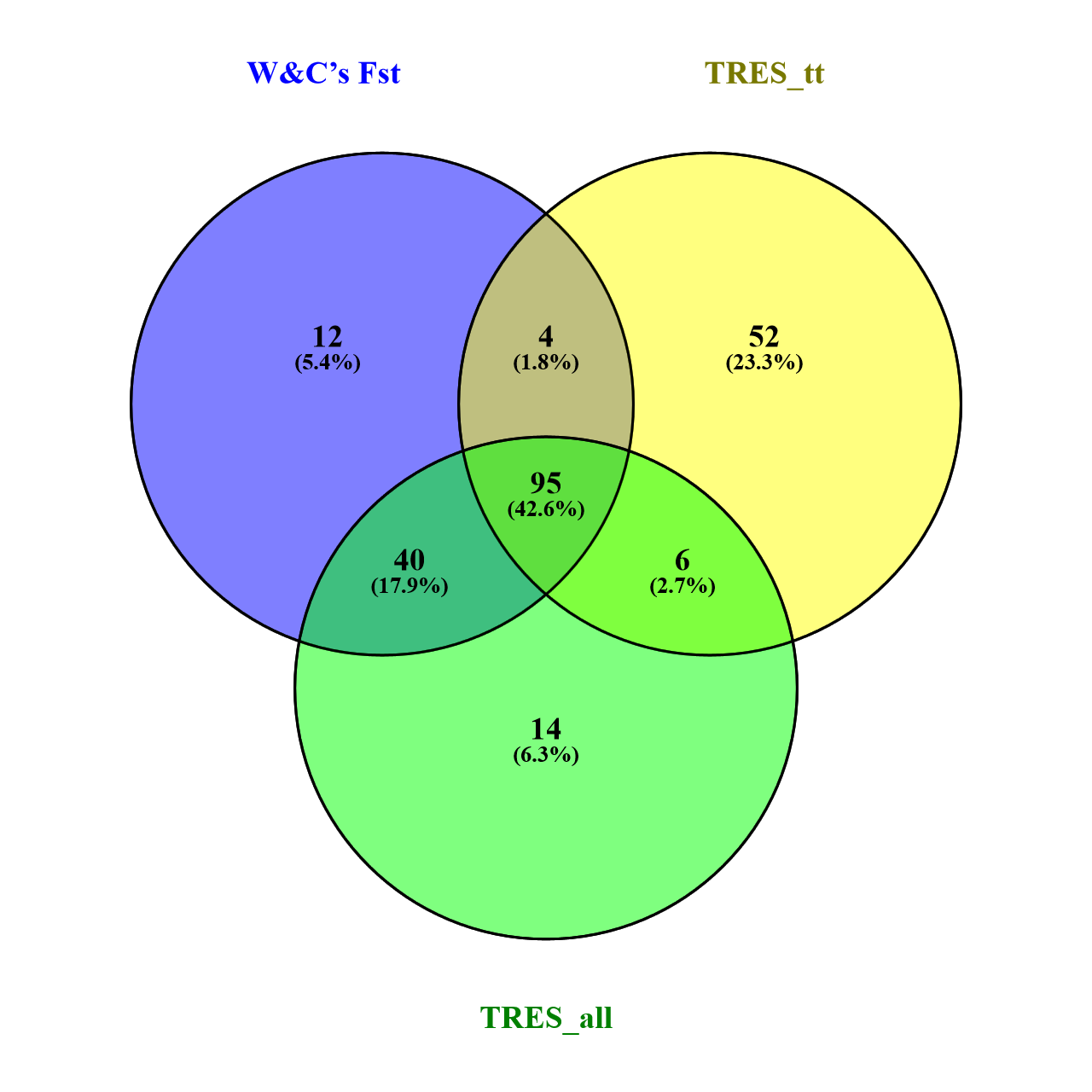

Supplement: S9 Fig — (TIFF) [file pone.0226179.s009.tiff]

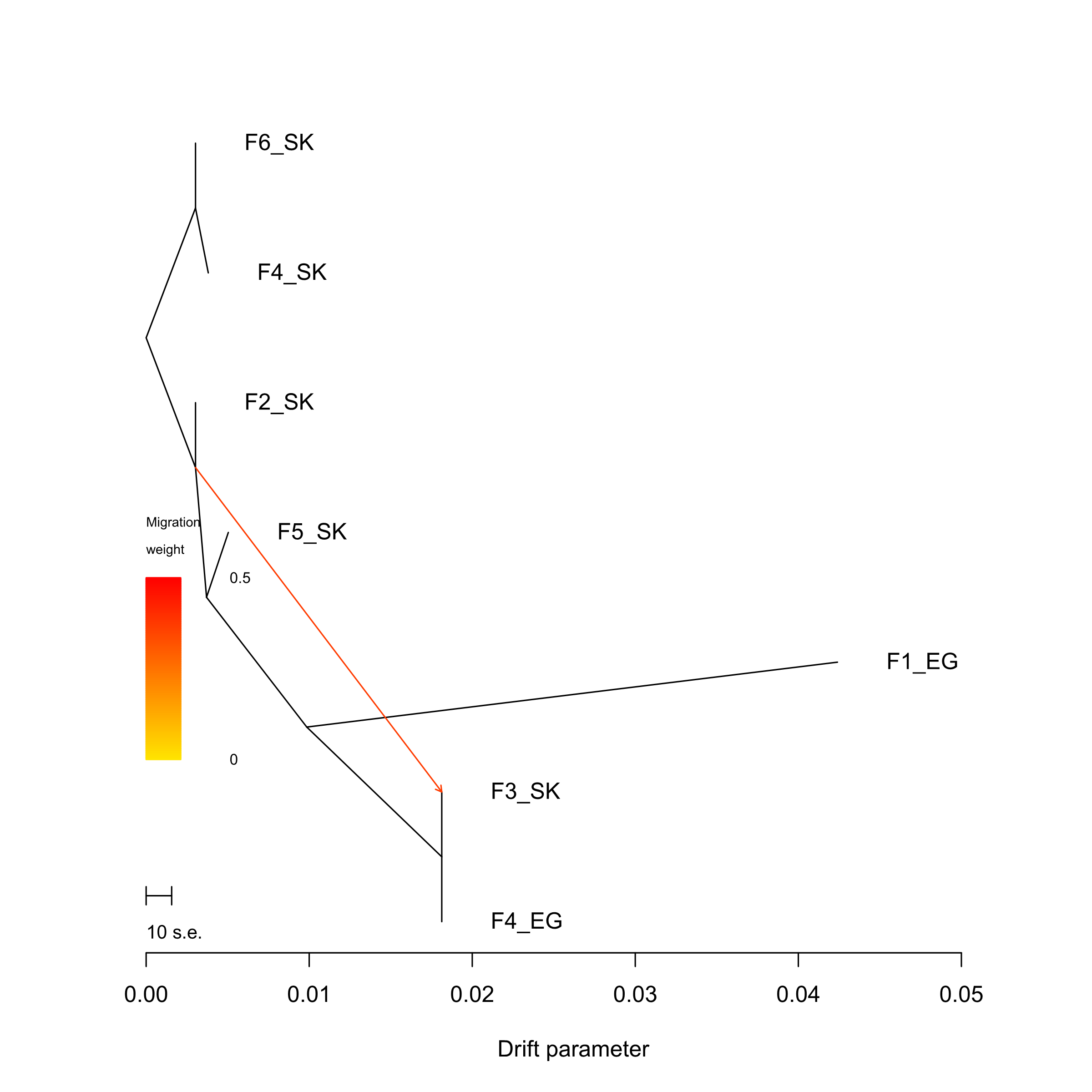

Supplement: S10 Fig — (TIFF) [file pone.0226179.s010.tiff]
