# Supplementary material for: Analysis of genome-wide DNA arrays reveals the genomic population structure and diversity in autochthonous Greek goat breeds
Source: PLoS One. 2019 Dec 12;14(12):e0226179. doi: 10.1371/journal.pone.0226179 (PMC6907847; doi:10.1371/journal.pone.0226179)
Supplement: S1 Table — (DOCX) [file pone.0226179.s011.docx]

**S1 Table. Information on farms, sampling locations and number of animals analyzed per breed.**

| **Farm** | **Samples** | **Breed** | **Samples** | **Region** | **Longitude** | **Latitude** |
| --- | --- | --- | --- | --- | --- | --- |
| Farm1 | EG1-EG15 | Eghoria | 15 | Tsepelovo | 39.895800 | 20.822658 |
| Farm2 | SK1-SK10 | Skopelos | 10 | Skopelos | 39.091693 | 23.718269 |
| Farm3 | SK11-SK20 | Skopelos | 10 | Skopelos | 39.090588 | 23.718976 |
| Farm4 | EG16-EG32 | Eghoria | 17 | Vassilika | 40.501167 | 23.116836 |
|  | SK21-SK28 | Skopelos | 8 |  |  |  |
| Farm5 | SK29-SK31 | Skopelos | 3 | Alonnisos | 39.213876 | 23.890877 |
| Farm6 | SK32-SK40 | Skopelos | 9 | Skopelos | 39.118086 | 23.723956 |
