# Supplementary material for: Analysis of genome-wide DNA arrays reveals the genomic population structure and diversity in autochthonous Greek goat breeds
Source: PLoS One. 2019 Dec 12;14(12):e0226179. doi: 10.1371/journal.pone.0226179 (PMC6907847; doi:10.1371/journal.pone.0226179)
Supplement: S2 Table — (DOCX) [file pone.0226179.s012.docx]

**S2 Table. Quality control of caprine Single Nucleotide Polymorphisms (SNPs).**

|  | Goat SNP50 BeadChip |
| --- | --- |
| Total number of SNPs | **53,347** |
| Call frequency (call rate) (<0.98) | 677 |
| Minor allele frequency (<1%) | 405 |
| HWE (≤1,0E-6) | 30 |
| X-linked SNPs / No chromosomal coordinates | 3,394 |
| Total SNPs remained | **48,841** |
