# Supplementary material for: Analysis of genome-wide DNA arrays reveals the genomic population structure and diversity in autochthonous Greek goat breeds
Source: PLoS One. 2019 Dec 12;14(12):e0226179. doi: 10.1371/journal.pone.0226179 (PMC6907847; doi:10.1371/journal.pone.0226179)
Supplement: S3 Table — (DOCX) [file pone.0226179.s013.docx]

**S3 Table. Estimates of ancestral effective population size (Ne) over past generations.**

| Generations ago | Eghoria | Skopelos |
| --- | --- | --- |
| 13 | 96 | 127 |
| 15 | 109 | 141 |
| 17 | 123 | 155 |
| 20 | 140 | 174 |
| 23 | 161 | 196 |
| 27 | 186 | 219 |
| 32 | 213 | 249 |
| 38 | 249 | 283 |
| 45 | 294 | 327 |
| 54 | 345 | 379 |
| 66 | 417 | 439 |
| 80 | 499 | 510 |
| 98 | 599 | 610 |
| 121 | 729 | 727 |
| 150 | 902 | 876 |
| 187 | 1,096 | 1,053 |
| 234 | 1,348 | 1,266 |
| 293 | 1,664 | 1,521 |
| 367 | 1,993 | 1,821 |
| 454 | 2,312 | 2,125 |
| 553 | 2,649 | 2,461 |
| 658 | 3,037 | 2,769 |
| 758 | 3,303 | 3,034 |
| 845 | 3,712 | 3,247 |
| 914 | 3,481 | 3,307 |
| 959 | 3,659 | 3,391 |
