# Supplementary material for: Analysis of genome-wide DNA arrays reveals the genomic population structure and diversity in autochthonous Greek goat breeds
Source: PLoS One. 2019 Dec 12;14(12):e0226179. doi: 10.1371/journal.pone.0226179 (PMC6907847; doi:10.1371/journal.pone.0226179)
Supplement: S5 Table — Genes located within ±100kb of the identified SNP or nearby genes of the identified SNPs are presented in italics. Texts in bold indicate the 95 common SNPs identified among the three methods. CHR: chromosome, kb: kilo base pair; SNP: Single nucleotide polymorphism. (DOCX) [file pone.0226179.s015.docx]

**Table S5 List of the 155 SNPs identified with the TRES software, evaluated in 64 individuals (TRES_all methodology).** Genes located within ±100kb of the identified SNPs or nearby genes of the identified SNPs are presented in italics. Texts in bold indicate the 95 common SNPs identified among the three methods. CHR: chromosome, kb: kilo base pair; SNP: Single Nucleotide Polymorphism.

| **SNP** | **CHR** | **SNP position** | **Gene(s) within ±100kb** | **Nearby gene(s)** |
| --- | --- | --- | --- | --- |
| snp26936-scaffold285-1097883 | 1 | 114939106 | *MBNL1* |  |
| snp46716-scaffold65-3473747 | 1 | 106001784 | *B3GALNT1, PPM1L, LOC108636207* |  |
| **snp33502-scaffold393-936881** | 2 | 66768518 | *-* | *DDX18, LOC102175338* |
| snp10697-scaffold138-732987 | 2 | 104933092 | *SLC38A11* |  |
| snp10714-scaffold138-1382399 | 2 | 104283680 | *-* | *FIGN, GRB14* |
| snp20424-scaffold202-2485175 | 2 | 32261918 | *-* | *LOC102185126, FN1* |
| snp38063-scaffold474-1405504 | 2 | 1405504 | *-* | *LOC108638512, IGSF21* |
| snp47657-scaffold670-229589 | 2 | 123727230 | *-* | *LOC102181546, LOC108638390* |
| snp7441-scaffold127-2638597 | 2 | 109461964 | *BBS5, LRP2, KLHL41* |  |
| **snp7513-scaffold127-5800510** | 2 | 112623877 | *RAPGEF4* |  |
| **snp10655-scaffold1378-189252** | 3 | 107422236 | *FCRL3, FCRL1, LOC102186680, LOC102186389* |  |
| **snp10666-scaffold1378-578833** | 3 | 107811817 | *KIRREL, LOC102180713* |  |
| **snp22459-scaffold222-1102516** | 3 | 37322030 | *PATJ, LOC102188010, LOC102183201, LOC102188186* |  |
| **snp29241-scaffold315-430267** | 3 | 9672353 | *-* | *C3H1orf94, GJB5* |
| snp36347-scaffold4353-70183 | 3 | 116630641 | *RXRG, LMX1A* |  |
| snp43619-scaffold582-1252502 | 3 | 36766020 | *TRNAQ-CUG, TM2D1, LOC102189087, LOC102183753* |  |
| **snp47963-scaffold675-4169261** | 3 | 29403522 | *LEXM, DHCR24, LOC108634584, TMEM61, BSND, USP24* |  |
| snp54320-scaffold83-1899178 | 3 | 74836057 | *DPYD* |  |
| snp24978-scaffold257-374255 | 3 | 62050007 | *LPAR3, MCOLN2, LOC106501954, MCOLN3* |  |
| **snp44372-scaffold603-4456529** | 4 | 11666429 | *-* | *CNTNAP2, TRNAS-GGA* |
| snp15283-scaffold1629-677846 | 4 | 105754797 | *-* | *TAC1, LOC102185057* |
| snp44287-scaffold603-795787 | 4 | 15327171 | *CLEC5A, LOC102178239, LOC102180188, LOC102178508, PRSS37, TAS2R5, TAS2R4, TAS2R3, SSBP1* |  |
| snp39040-scaffold494-6742299 | 4 | 73621110 | *ATXN7L1, LOC102170111, RINT1, PUS7* |  |
| **snp33395-scaffold392-1619441** | 5 | 55101337 | *AVIL, TSFM, METTL21B, METTL1, LOC108636103, LOC102170758, MARCH9, CDK4, TSPAN31, AGAP2, OS9, LOC108636010, B4GALNT1, SLC26A110, LOC108636011, ARHGF25* |  |
| **snp10787-scaffold1384-759026** | 5 | 107094012 | *WNT5B, FBXL14, ADIPOR2, CACNA2D4, LRTM2* | *WNT5B, ADIPOR2* |
| **snp271-scaffold1007-133258** | 5 | 73216593 | *LOC108636110, LOC106503978, LOC108636024, LOC102172252, LOC102172429, LOC102171493, LOC108636132, LOC102177504* |  |
| **snp273-scaffold1007-204113** | 5 | 73145738 | *LOC102170968, LOC108636023, LOC102171143, LOC108636108, LOC108636109, LOC108636110, LOC106503978, LOC108636024, LOC102172252, LOC102172428* |  |
| **snp28712-scaffold310-313359** | 5 | 62372487 | *ANKS1B, FAM71C, LOC102172242, LOC108636089* |  |
| **snp33401-scaffold392-1890664** | 5 | 54830114 | *ATP23* |  |
| **snp34488-scaffold405-2384232** | 5 | 11246408 | *LOC108636092* |  |
| **snp36656-scaffold443-1104385** | 5 | 16638543 | *LOC102169555* |  |
| snp8935-scaffold1322-262746 | 5 | 85616577 | *-* | *LOC102175101, TRNAK-UUU* |
| snp7619-scaffold1273-72450 | 6 | 38482775 | *-* | *LOC102178056, LCORL* |
| **snp58078-scaffold94-4855213** | 6 | 67753278 | *ZAR1, SLA1N2, SLC10A4, FRYL* |  |
| **snp16117-scaffold1698-297420** | 6 | 112692374 | *-* | *LDB2, QDPR* |
| **snp26757-scaffold281-954177** | 6 | 35289058 | *MMRN1, SNCA, LOC108636214* |  |
| **snp30824-scaffold340-1241741** | 6 | 12293091 | *CAMK2D* |  |
| **snp42243-scaffold553-586410** | 6 | 19439848 | *TBCK, NPNT* |  |
| snp4467-scaffold1143-598605 | 6 | 90586915 | *PARM1, LOC106502208* |  |
| snp16112-scaffold1698-77702 | 6 | 112912092 | *-* | *LDB2, QDPR* |
| snp17106-scaffold1789-40192 | 6 | 47881611 | *-* | *LOC102171416, LOC102171698* |
| **snp5698-scaffold12-516413** | 7 | 83774644 | *MARCH3, LOC102174382, C7H5orf63* |  |
| **snp55332-scaffold853-1414986** | 7 | 85706142 | *ISOC1* |  |
| **snp10027-scaffold1356-1806287** | 7 | 48872935 | *SLC6A7, ARSI, CAMKA2A, CDX1, PDGFRB* |  |
| **snp1801-scaffold105-1044230** | 7 | 35564393 | *LOC106502366* |  |
| **snp21526-scaffold210-1174425** | 7 | 104042315 | *ELL, GDF15, LRRC25, SSBP4, ISYNA1, FKBP8, KXD1, UBA52* |  |
| **snp23243-scaffold2321-341593** | 7 | 12699429 | *RGMB* |  |
| **snp29821-scaffold323-2825818** | 7 | 65138526 | *FSTL4* |  |
| **snp30585-scaffold339-1181881** | 7 | 42583219 | *SGCD* |  |
| **snp30586-scaffold339-1217081** | 7 | 42618419 | *SGCD* |  |
| **snp30616-scaffold339-2434023** | 7 | 43835361 | *LOC106502343* |  |
| **snp36625-scaffold441-1419110** | 7 | 95640993 | *ELAVL3, TSPAN16, RAB3D, TMEM205, CCDC159, PLPPR2, SWSAP1, EPOR, RGL3, CCDC151, PRKCSH, ZNF653, ECSIT* |  |
| **snp55334-scaffold853-1510622** | 7 | 85801778 | *-* | *ISOC1, ADAMTS19* |
| snp8842-scaffold1317-953886 | 7 | 32337542 | *-* | *LOC102187402, LOC102187131* |
| **snp17056-scaffold178-6924** | 8 | 110800231 | *RNASEH1, ADI1, TRAPPC12, TSSC1* |  |
| **snp31919-scaffold356-5375114** | 8 | 103713352 | *TNFSF15, TNFSF8, LOC102178728* |  |
| snp46348-scaffold64-1373506 | 8 | 33206301 | *-* | *LOC108636662, LOC102173935* |
| snp31870-scaffold356-3145635 | 8 | 101483873 | *PTBP3, LOC106501724, TRNAC-GCA, HSDL2, KIAA1958* |  |
| **snp8864-scaffold1318-886389** | 9 | 5822706 | *IRAK1BP1, PHIP* |  |
| **snp22975-scaffold2290-1529796** | 9 | 73365307 | *UST* |  |
| snp29422-scaffold318-2042768 | 9 | 51727599 | *-* | *LOC102189401, TBX18* |
| snp32006-scaffold359-1698144 | 9 | 11358009 | *RNF146, RSPO3, ECHDC1, TRNAS-AGA* |  |
| **snp43752-scaffold588-885293** | 9 | 13941545 | *NKAIN2* |  |
| snp45518-scaffold620-3479179 | 9 | 54837725 | *LAMA2* |  |
| snp18244-scaffold185-18663511 | 10 | 68199511 | *C10H15orf54* |  |
| snp11517-scaffold1420-232400 | 10 | 97146550 | *CCDC112, LOC102182794, PGGT1B, TRIM36* |  |
| **snp33776-scaffold397-2193850** | 10 | 33916790 | *-* | *LOC102173663, OTX2* |
| **snp56398-scaffold886-34240** | 10 | 6809713 | *-* | *LOC108636851,LOC108636903* |
| **snp27474-scaffold293-70609** | 11 | 12763575 | *DYSF* |  |
| snp33908-scaffold4-982201 | 11 | 93324955 | *LOC102178634, LOC102178368, LOC102178083, LOC102177808, LOC102177535, LOC102177251, LOC102181033, LOC102176137, LOC102175865, LOC102176702, LOC102176434, LOC108637119, LOC102179209, LOC108637121, LOC102182883* |  |
| snp40644-scaffold52-2553548 | 11 | 29510240 | *KCNK12, MSH2, LOC106502612* |  |
| **snp45261-scaffold618-1296976** | 11 | 18215606 | *-* | *LOC102182881, LOC102183425* |
| **snp52870-scaffold793-645929** | 11 | 47614584 | *FABP1, SMYD1, KRCC1, LOC102187869, LOC102188142, CD8B* |  |
| snp9725-scaffold135-865412 | 11 | 23636212 | *-* | *LOC102186970, TRNAG-UCC* |
| **snp3130-scaffold1095-1777706** | 12 | 31319637 | *NDFIP2* |  |
| **snp3199-scaffold1095-4607609** | 12 | 28489734 | *TRNAC-GCA* |  |
| **snp46526-scaffold644-433228** | 12 | 4041103 | *ARGLU1, EFNB2* |  |
| **snp49558-scaffold707-845015** | 12 | 4497015 | *-* | *LOC108637268,TRNAS-GGA* |
| snp50253-scaffold717-7851732 | 12 | 28236673 | *-* | *SLITRK1, TRNAC-GCA* |
| snp52079-scaffold77-147551 | 12 | 75947890 | *OLFM4, LOC108637282* |  |
| snp6850-scaffold125-2477080 | 12 | 45842258 | *-* | *LOC102173479, PCDH9* |
| snp32392-scaffold369-263942 | 13 | 8004757 | *MACROD2, TRNAC-GCA* |  |
| **snp49036-scaffold7-3156990** | 13 | 29235004 | *FAM171A1* |  |
| **snp13043-scaffold150-2812373** | 13 | 48727169 | *BMP2* |  |
| **snp23764-scaffold24-1322909** | 13 | 24413520 | *KIAA1217, LOC106502743, LOC108637381* |  |
| **snp58666-scaffold956-583320** | 13 | 69639236 | *LPIN3, EMILIN3, TRNAE-CUC, CHD6* |  |
| snp56093-scaffold875-1767141 | 13 | 13733085 | *-* | *TRNAE-UUC, TRNAS-GGA* |
| **snp11119-scaffold14-2120966** | 14 | 69372832 | *MYC, LOC102186225* |  |
| **snp10979-scaffold1394-9820** | 15 | 3788090 | *LOC102186788, LOC102186505, LOC102186235, LOC102178290, LOC102185943, LOC102185678, LOC102185387, LOC102185108, LOC102184831, LOC102184545, LOC102178000, LOC102184269, LOC102183988, LOC102183711, LOC108637600, LOC106502922, LOC106501735* |  |
| snp36734-scaffold445-2068057 | 15 | 32799035 | *LOC102186232, LOC102174230, LOC102185483, LOC102173954, LOC102173403, LOC108637633, LOC102184740, LOC102184170, LOC102184454, LOC102173107, TRIM68* |  |
| snp42257-scaffold5554-71655 | 15 | 63266107 | *-* | *C15H11orf87, DDX10* |
| **snp5289-scaffold1183-1232002** | 15 | 24197669 | *METTL15, KIF18A* |  |
| snp14809-scaffold1599-344370 | 16 | 47612243 | *-* | *AJAP1, C16H1orf174* |
| snp4926-scaffold1167-25526 | 16 | 11518091 | *RGS21* |  |
| **snp52577-scaffold785-78925** | 16 | 71491257 | *HHAT* |  |
| **snp8588-scaffold131-488644** | 16 | 57929033 | *SEC16B, LOC102182333* |  |
| **snp8590-scaffold131-590370** | 16 | 57827307 | *SEC16B* |  |
| snp17021-scaffold1776-238781 | 16 | 65393230 | *HMCN1* |  |
| snp37108-scaffold4500-191594 | 16 | 47227493 | *AJAP1* |  |
| **snp35584-scaffold428-3644394** | 17 | 31015420 | *FNIP2, C17H4orf45* |  |
| **snp22681-scaffold225-2453071** | 18 | 32283098 | *-* | *LOC102172836, TRNAC-ACA* |
| snp18714-scaffold1890-461100 | 18 | 53368328 | *ZNF112, ZNF235, ZNF285, LOC102168853, ZNF180, LOC102169126, CEACAM20, LOC102169412, IGSF23* |  |
| **snp29471-scaffold3193-206680** | 18 | 54727121 | *HIF3A, PPP5C, LOC108638115, PNMAL1, LOC102169127, CCDC8, PNMAL2, TRNAA-AGC, CALM3, PTGIR, GNG8, DACT3* |  |
| snp3081-scaffold1093-1662245 | 18 | 30310592 | *LOC102172291* |  |
| **snp40193-scaffold512-1535132** | 18 | 16674389 | *-* | *TRNAC-GCA, LOC106503092* |
| **snp45426-scaffold62-3872419** | 20 | 31082908 | *NNT* |  |
| **snp49800-scaffold711-1045536** | 20 | 20243209 | *PDE4D* |  |
| snp12295-scaffold1460-979199 | 21 | 57833966 | *PRIMA1, UNC79* |  |
| snp14957-scaffold160-2042140 | 21 | 26860755 | *TMC3, LOC108638535, MCEE, LOC102181633, LOC102177475* |  |
| snp14978-scaffold160-2905881 | 21 | 27724496 | *LOC102183271, NSMCE3, FAM189A1* |  |
| **snp32586-scaffold374-29855** | 21 | 21346669 | *CRTC3, IQGAP1* |  |
| **snp38814-scaffold492-2146782** | 21 | 39231124 | *-* | *LOC108638490, PRKD1* |
| **snp50303-scaffold72-41841** | 21 | 7808696 | *LOC108638451* |  |
| **snp51448-scaffold754-481947** | 21 | 20834768 | *ZNF710, IDH2, LOC102186633, SEMA4B, CIB1, GDPGP1, NGRN* |  |
| **snp57122-scaffold91-477178** | 21 | 4049239 | *-* | *GABRA5, LOC102178769* |
| **snp57235-scaffold911-1204549** | 21 | 15959450 | *LOC106503345, KLHL25* |  |
| **snp7146-scaffold1265-72004** | 21 | 17693088 | *TRNAG-UCC* |  |
| **snp7153-scaffold1265-368894** | 21 | 17396198 | *-* | *AGBL1, TRNAG-UCC* |
| snp57168-scaffold91-2506997 | 21 | 2019420 | *ATP10A, LOC102191179* |  |
| snp19109-scaffold1921-51786 | 21 | 32652580 | *LINGO1, ODF3L1, CSPG4, SNX33, SNUPN, LOC102190173, IMP3, PTPN9* |  |
| **snp2949-scaffold109-1754220** | 22 | 8404395 | *-* | *TRNAS-GGA, TRNAQ-CUG* |
| snp2955-scaffold109-1993557 | 22 | 8643732 | *-* | *TRNAS-GGA, TRNAQ-CUG* |
| snp15346-scaffold163-2748091 | 22 | 38293677 | *-* | *SYNPR, CADPS* |
| snp10259-scaffold1368-2187818 | 23 | 41127485 | *ZBTB9, SYNGAP1, CUTA, PHF1, KIFC1, LOC102183375, LOC102168498, DAXX, ZBTB22, TAPBP, RGL2, PFDN6, WDR46* |  |
| **snp11458-scaffold1417-946413** | 23 | 16256899 | *FARS2, LYRM4, LOC102169818* |  |
| **snp39678-scaffold505-474676** | 23 | 36272066 | *DNAH8, GLO1, BTBD9* |  |
| snp20296-scaffold2009-4422 | 23 | 48440896 | *LOC102180545, KHDRBS2* |  |
| **snp9699-scaffold1349-495354** | 24 | 5803577 | *LOC102180814* |  |
| **snp10677-scaffold1379-313938** | 24 | 12707725 | *LOC102170373, SYT4* |  |
| snp37147-scaffold451-1393943 | 24 | 13617522 | *-* | *LOC108633764, LOC102171118* |
| **snp54916-scaffold84-1663231** | 24 | 3258805 | *-* | *ZNF516, TSHZ1* |
| **snp7659-scaffold1277-264730** | 24 | 49457980 | *LOC108633809, LIPG* | *LIPG, LOC108633809* |
| snp7669-scaffold1277-653936 | 24 | 49068774 | *DYM* |  |
| snp30489-scaffold336-1456468 | 25 | 9399905 | *TEKT5, NUBP1, TVP23A, CIITA, DEXI* |  |
| **snp38343-scaffold485-862961** | 25 | 16554591 | *CLEC19A, TRNAK-CUU, SYT17* |  |
| **snp43024-scaffold570-1172048** | 25 | 6941744 | *-* | *RBFOX1, TMEM114* |
| **snp15063-scaffold161-373737** | 26 | 14520936 | *KCNK18, VAX1, SHTN1* |  |
| **snp31449-scaffold3488-54845** | 26 | 48980676 | *ZWINT* |  |
| **snp42278-scaffold556-861145** | 26 | 8347596 | *CHST15, CPXM2* |  |
| snp55137-scaffold847-1077269 | 26 | 7005877 | *-* | *LOC102180995, CTBP2* |
| **snp55159-scaffold847-2003062** | 26 | 6080084 | *C26H10orf90, ADAM12* |  |
| **snp44504-scaffold604-3249879** | 27 | 13721478 | *-* | *KCNU1, LOC102184589* |
| **snp30300-scaffold333-3618181** | 27 | 9466438 | *SFRP1, LOC102185808* |  |
| snp30322-scaffold333-4592195 | 27 | 10440452 | *C27H8orf4, IDO2* |  |
| **snp30734-scaffold34-1571279** | 27 | 3219984 | *LOC102174361* |  |
| **snp44450-scaffold604-977126** | 27 | 11448725 | *LOC102191489, ADAM9, LOC102191206* |  |
| **snp44483-scaffold604-2371901** | 27 | 12843500 | *-* | *TRNAE-UUC, LOC102185144* |
| **snp54865-scaffold838-4305667** | 28 | 16059681 | *USP54, LOC102187373, FUT11, SEC24C, SYNPO2L, MYOZ1, PPP3CB* |  |
| **snp57597-scaffold922-153439** | 28 | 546413 | *-* | *LOC102169834, CXCL12* |
| snp14780-scaffold1596-296344 | 29 | 1362123 | *MTNR1B, LOC102176586* |  |
| **snp17567-scaffold182-274423** | 29 | 43971595 | *POLA2, CDC43EP2, DPF2, TIGD3, SLC25A45, FRMD8, TRNAS-GGA* |  |
| **snp227-scaffold1006-227193** | 29 | 7986537 | *TMEM135, LOC102191495, FZD4* |  |
| **snp48271-scaffold683-1146836** | 29 | 11630471 | *DLG2* |  |
| **snp53108-scaffold799-3285503** | 29 | 35315323 | *TMEM45B, NFRKB, PRDM10* |  |
| snp2830-scaffold1083-377270 | 29 | 26036694 | *LOC102170028, LOC102188475, LOC102188745, LOC102189009, LOC102170300, LOC102170584, LOC102170855, LOC102171132, LOC102171408, LOC102171690, LOC102171954, LOC102189280, LOC102189836, LOC106503724* |  |
