# Supplementary material for: Analysis of genome-wide DNA arrays reveals the genomic population structure and diversity in autochthonous Greek goat breeds
Source: PLoS One. 2019 Dec 12;14(12):e0226179. doi: 10.1371/journal.pone.0226179 (PMC6907847; doi:10.1371/journal.pone.0226179)
Supplement: S6 Table — Genes located within ±100kb of the identified SNP or nearby genes of the identified SNPs are presented in italics. Texts in bold indicate the 95 common SNPs identified among the three methods. CHR: chromosome, kb: kilo base pair; SNP: Single nucleotide polymorphism. (DOCX) [file pone.0226179.s016.docx]

**Table S6 List of the 157 SNPs identified with the TRES software, by splitting the dataset into training and test populations (TRES_tt methodology).** Genes located within ±100kb of the identified SNPs or nearby genes of the identified SNPs are presented in italics. Texts in bold indicate the 95 common SNPs identified among the three methods. CHR: chromosome, kb: kilo base pair; SNP: Single Nucleotide Polymorphism.

| **SNP** | **CHR** | **SNP position** | **Gene(s) within ±100kb** | **Nearby gene(s)** |
| --- | --- | --- | --- | --- |
| snp13288-scaffold1510-626012 | 1 | 80678366 | *DGKG* |  |
| snp23015-scaffold230-1160925 | 1 | 132030081 | *STAG1* |  |
| snp28573-scaffold306-641061 | 1 | 62708603 | *-* | *LOC102189521, TRNAS-GGA* |
| snp40295-scaffold514-1162885 | 1 | 140415818 | *DSCAM, LOC102179816* |  |
| snp40528-scaffold519-1333499 | 1 | 77608390 | *-* | *TP63, LOC102178860* |
| snp2008-scaffold1059-835165 | 2 | 130774690 | *MFSD6, LOC108638407, NEMP2, NAB1* |  |
| **snp33502-scaffold393-936881** | 2 | 66768518 | *-* | *DDX18, LOC102175338* |
| snp51387-scaffold752-14780 | 2 | 58013150 | *LOC108633415* |  |
| **snp7513-scaffold127-5800510** | 2 | 112623877 | *RAPGEF4* |  |
| **snp10655-scaffold1378-189252** | 3 | 107422236 | *FCRL3, FCRL1, LOC102186680, LOC102186389* |  |
| **snp10666-scaffold1378-578833** | 3 | 107811817 | *KIRREL, LOC102180713* |  |
| **snp22459-scaffold222-1102516** | 3 | 37322030 | *PATJ, LOC102188010, LOC102183201, LOC102188186* |  |
| snp23580-scaffold238-392830 | 3 | 100189953 | *GOLPH3L, LOC102184486, LOC102184771, HORMAD1, CTSS, LOC102179724, CTSK, ARNT* |  |
| snp24978-scaffold257-374255 | 3 | 62050007 | *LPAR3, MCOLN2, LOC106501954, MCOLN3* |  |
| **snp29241-scaffold315-430267** | 3 | 9672353 | *-* | *C3H1orf94, GJB5* |
| snp46910-scaffold654-1771372 | 3 | 34605119 | *FGGY, LOC102191667, HOOK1* |  |
| **snp47963-scaffold675-4169261** | 3 | 29403522 | *LEXM, DHCR24, LOC108634584, TMEM61, BSND, USP24* |  |
| snp56492-scaffold89-853164 | 3 | 10353865 | *SFPQ, LOC102168967, ZMYM4, LOC102183387* |  |
| snp10844-scaffold1386-868744 | 4 | 100338953 | *TRNAC-ACA* |  |
| snp15283-scaffold1629-677846 | 4 | 105754797 | *-* | *TAC1, LOC102185057* |
| snp32092-scaffold362-492730 | 4 | 92298257 | *-* | *TWISTNB, FERD3L* |
| snp44287-scaffold603-795787 | 4 | 15327171 | *CLEC5A, LOC102178239, LOC102180188, LOC102178508, PRSS37, TAS2R5, TAS2R4, TAS2R3, SSBP1* |  |
| snp44301-scaffold603-1424112 | 4 | 14698846 | *LOC102173285, LOC102172430, LOC102169377, LOC102171862, LOC102168816, LOC102171594, LOC102171323, LOC102170222, LOC102169952, LOC108635878, LOC108635879, LOC108635880, LOC108635881, LOC108635882, LOC108635830, LOC108635833* |  |
| snp44365-scaffold603-4115280 | 4 | 12007678 | *-* | *CNTNAP2, TRNAS-GGA* |
| **snp44372-scaffold603-4456529** | 4 | 11666429 | *-* | *CNTNAP2, TRNAS-GGA* |
| **snp10787-scaffold1384-759026** | 5 | 107094012 | *WNT5B, FBXL14, ADIPOR2, CACNA2D4, LRTM2* | *WNT5B, ADIPOR2* |
| **snp271-scaffold1007-133258** | 5 | 73216593 | *LOC108636110, LOC106503978, LOC108636024, LOC102172252, LOC102172429, LOC102171493, LOC108636132, LOC102177504* |  |
| **snp273-scaffold1007-204113** | 5 | 73145738 | *LOC102170968, LOC108636023, LOC102171143, LOC108636108, LOC108636109, LOC108636110, LOC106503978, LOC108636024, LOC102172252, LOC102172428* |  |
| **snp28712-scaffold310-313359** | 5 | 62372487 | *ANKS1B, FAM71C, LOC102172242, LOC108636089* |  |
| **snp33395-scaffold392-1619441** | 5 | 55101337 | *AVIL, TSFM, METTL21B, METTL1, LOC108636103, LOC102170758, MARCH9, CDK4, TSPAN31, AGAP2, OS9, LOC108636010, B4GALNT1, SLC26A110, LOC108636011, ARHGF25* |  |
| **snp33401-scaffold392-1890664** | 5 | 54830114 | *ATP23* |  |
| **snp34488-scaffold405-2384232** | 5 | 11246408 | *LOC108636092* |  |
| **snp36656-scaffold443-1104385** | 5 | 16638543 | *LOC102169555* |  |
| snp40714-scaffold521-537618 | 5 | 27684179 | *ACVR1B, ACVRL1, ANKRD33, FIGNL2, SCN8A* |  |
| snp51022-scaffold740-86044 | 5 | 88940487 | *LOC108636134, LOC102172130, AEBP2* |  |
| snp12606-scaffold148-4489143 | 6 | 33328019 |  |  |
| **snp16117-scaffold1698-297420** | 6 | 112692374 | *-* | *LDB2, QDPR* |
| snp26739-scaffold281-267352 | 6 | 34602233 | *-* | *TRNA-GCA, CCSER1* |
| **snp26757-scaffold281-954177** | 6 | 35289058 | *MMRN1, SNCA, LOC108636214* |  |
| snp26780-scaffold281-1934289 | 6 | 36269170 | *TIGD2, FAM13A* |  |
| **snp30824-scaffold340-1241741** | 6 | 12293091 | *CAMK2D* |  |
| snp41590-scaffold541-929571 | 6 | 53071152 |  | *TRNAW-CCA, TRNAC-GCA* |
| **snp42243-scaffold553-586410** | 6 | 19439848 | *TBCK, NPNT* |  |
| snp4408-scaffold1139-1361373 | 6 | 106426116 | *TRNAA-UGC* |  |
| snp49741-scaffold710-1002906 | 6 | 18384358 | *DKK2* |  |
| snp52931-scaffold795-773833 | 6 | 15059911 | *LOC108636272, PITX2* |  |
| **snp58078-scaffold94-4855213** | 6 | 67753278 | *ZAR1, SLA1N2, SLC10A4, FRYL* |  |
| snp59989-scaffold999-274745 | 6 | 1584320 |  | *LOC102175391, LOC102186848* |
| **snp10027-scaffold1356-1806287** | 7 | 48872935 | *SLC6A7, ARSI, CAMKA2A, CDX1, PDGFRB* |  |
| snp13790-scaffold1539-710641 | 7 | 105218786 |  |  |
| **snp1801-scaffold105-1044230** | 7 | 35564393 | *LOC106502366* |  |
| snp20073-scaffold2-2668014 | 7 | 89706196 | *GNG7, DIRAS1, SLC39A3, SGTA, THOP1, ZNF554, LOC102181027, LOC106502316, LOC102180305, TLE6, TLE2* |  |
| **snp21526-scaffold210-1174425** | 7 | 104042315 | *ELL, GDF15, LRRC25, SSBP4, ISYNA1, FKBP8, KXD1, UBA52* |  |
| snp21548-scaffold210-2183626 | 7 | 103033114 | *USHBP1, BABAM1, ANKLE1, ABHD8, TRNAK-CUU, MRPL34, DDA1, ANO8, GTPBP3, PLVAP, LOC106502257, BST2, LOC108636404, LOC108636449, MVB12A, TMEM221, NXNL1* |  |
| **snp23243-scaffold2321-341593** | 7 | 12699429 | *RGMB* |  |
| **snp29821-scaffold323-2825818** | 7 | 65138526 | *FSTL4* |  |
| **snp30585-scaffold339-1181881** | 7 | 42583219 | *SGCD* |  |
| **snp30586-scaffold339-1217081** | 7 | 42618419 | *SGCD* |  |
| **snp30616-scaffold339-2434023** | 7 | 43835361 | *LOC106502343* |  |
| snp33118-scaffold386-1508886 | 7 | 34800413 | *-* | *LOC102187131, MAT2B* |
| **snp36625-scaffold441-1419110** | 7 | 95640993 | *ELAVL3, TSPAN16, RAB3D, TMEM205, CCDC159, PLPPR2, SWSAP1, EPOR, RGL3, CCDC151, PRKCSH, ZNF653, ECSIT* |  |
| snp50279-scaffold718-867017 | 7 | 93354767 | *ADAMTS10, MYO1F, ZNF414, PRAM1, HNRNPM, MARCH2, RAB11B, ANGPTL4, KANK3* |  |
| **snp55332-scaffold853-1414986** | 7 | 85706142 | *ISOC1* |  |
| **snp55334-scaffold853-1510622** | 7 | 85801778 | *-* | *ISOC1, ADAMTS19* |
| **snp5698-scaffold12-516413** | 7 | 83774644 | *MARCH3, LOC102174382, C7H5orf63* |  |
| snp8832-scaffold1317-596475 | 7 | 32694953 | *-* | *LOC102187402, LOC102187131* |
| snp8842-scaffold1317-953886 | 7 | 32337542 | *-* | *LOC102187402, LOC102187131* |
| **snp17056-scaffold178-6924** | 8 | 110800231 | *RNASEH1, ADI1, TRAPPC12, TSSC1* |  |
| **snp31919-scaffold356-5375114** | 8 | 103713352 | *TNFSF15, TNFSF8, LOC102178728* |  |
| snp992-scaffold1026-127301 | 8 | 69364930 | *PWIL2, SLC39A14, PPP3CC* |  |
| **snp22975-scaffold2290-1529796** | 9 | 73365307 | *UST* |  |
| **snp43752-scaffold588-885293** | 9 | 13941545 | *NKAIN2* |  |
| **snp8864-scaffold1318-886389** | 9 | 5822706 | *IRAK1BP1, PHIP* |  |
| snp10203-scaffold1366-689257 | 10 | 47268020 | *UNC13C* |  |
| snp24664-scaffold251-1433721 | 10 | 43079810 | *ATP8B4, SLC27A2, HDC* |  |
| **snp33776-scaffold397-2193850** | 10 | 33916790 | *-* | *LOC102173663, OTX2* |
| **snp56398-scaffold886-34240** | 10 | 6809713 | *-* | *LOC108636851,LOC108636903* |
| snp10935-scaffold1392-450133 | 11 | 83539595 | *TRNAC-ACA* |  |
| snp170-scaffold1002-288198 | 11 | 50080695 | *DNAH6, SUCLG1, LOC102173197* |  |
| **snp27474-scaffold293-70609** | 11 | 12763575 | *DYSF* |  |
| **snp45261-scaffold618-1296976** | 11 | 18215606 | *-* | *LOC102182881, LOC102183425* |
| **snp52870-scaffold793-645929** | 11 | 47614584 | *FABP1, SMYD1, KRCC1, LOC102187869, LOC102188142, CD8B* |  |
| **snp3130-scaffold1095-1777706** | 12 | 31319637 | *NDFIP2* |  |
| **snp3199-scaffold1095-4607609** | 12 | 28489734 | *TRNAC-GCA* |  |
| snp39129-scaffold498-2005881 | 12 | 13583065 | *LOC108637288, LOC102187779, LOC108637249* |  |
| **snp46526-scaffold644-433228** | 12 | 4041103 | *ARGLU1, EFNB2* |  |
| **snp49558-scaffold707-845015** | 12 | 4497015 | *-* | *LOC108637268,TRNAS-GGA* |
| **snp13043-scaffold150-2812373** | 13 | 48727169 | *BMP2* |  |
| **snp23764-scaffold24-1322909** | 13 | 24413520 | *KIAA1217, LOC106502743, LOC108637381* |  |
| **snp49036-scaffold7-3156990** | 13 | 29235004 | *FAM171A1* |  |
| **snp58666-scaffold956-583320** | 13 | 69639236 | *LPIN3, EMILIN3, TRNAE-CUC, CHD6* |  |
| snp58774-scaffold959-1552261 | 13 | 43433610 | *LOC106502758* |  |
| **snp11119-scaffold14-2120966** | 14 | 69372832 | *MYC, LOC102186225* |  |
| snp23458-scaffold2365-122165 | 14 | 64940864 | *-* | *LOC102170064, XHX2* |
| snp51263-scaffold75-4682303 | 14 | 36442738 | *SAMD12, LOC106502867* |  |
| snp51290-scaffold75-5863585 | 14 | 35261456 | *MED30* |  |
| **snp10979-scaffold1394-9820** | 15 | 3788090 | *LOC102186788, LOC102186505, LOC102186235, LOC102178290, LOC102185943, LOC102185678, LOC102185387, LOC102185108, LOC102184831, LOC102184545, LOC102178000, LOC102184269, LOC102183988, LOC102183711, LOC108637600, LOC106502922, LOC106501735* |  |
| snp2056-scaffold1062-632905 | 15 | 46009669 | *SOX6* |  |
| **snp5289-scaffold1183-1232002** | 15 | 24197669 | *METTL15, KIF18A* |  |
| snp13988-scaffold1551-363791 | 16 | 8730077 | *-* | *TRNAK-UUU, CDC73* |
| **snp52577-scaffold785-78925** | 16 | 71491257 | *HHAT* |  |
| snp55081-scaffold845-566536 | 16 | 17539970 | *USH2A, LOC102172086* |  |
| **snp8588-scaffold131-488644** | 16 | 57929033 | *SEC16B, LOC102182333* |  |
| **snp8590-scaffold131-590370** | 16 | 57827307 | *SEC16B* |  |
| snp8646-scaffold131-2943126 | 16 | 55474551 | *TNR* |  |
| snp21202-scaffold207-4027158 | 17 | 16304105 | *CUX2* |  |
| **snp35584-scaffold428-3644394** | 17 | 31015420 | *FNIP2, C17H4orf45* |  |
| snp55421-scaffold858-1896427 | 17 | 9878290 | *LOC102190983, LOC102186893, SLC8B1, TPCN1, IQCD, RITA1, DDX54, CFAP73* |  |
| **snp22681-scaffold225-2453071** | 18 | 32283098 | *-* | *LOC102172836, TRNAC-ACA* |
| **snp29471-scaffold3193-206680** | 18 | 54727121 | *HIF3A, PPP5C, LOC108638115, PNMAL1, LOC102169127, CCDC8, PNMAL2, TRNAA-AGC, CALM3, PTGIR, GNG8, DACT3* |  |
| snp37903-scaffold467-1402210 | 18 | 29804500 | *-* | *LOC102172002, LOC102172291* |
| **snp40193-scaffold512-1535132** | 18 | 16674389 | *-* | *TRNAC-GCA, LOC106503092* |
| snp59703-scaffold99-318969 | 18 | 14531916 | *ZCCHC14, JPH3, KLHDC4* |  |
| snp3324-scaffold1101-1054879 | 19 | 54465175 | *LOC102184279, LOC102184758, ST6GALNAC2, LOC102184471, PRCD, CYGB, RHBDF2, AANAT, UBE2O* |  |
| **snp45426-scaffold62-3872419** | 20 | 31082908 | *NNT* |  |
| **snp49800-scaffold711-1045536** | 20 | 20243209 | *PDE4D* |  |
| **snp32586-scaffold374-29855** | 21 | 21346669 | *CRTC3, IQGAP1* |  |
| **snp38814-scaffold492-2146782** | 21 | 39231124 | *-* | *LOC108638490, PRKD1* |
| **snp50303-scaffold72-41841** | 21 | 7808696 | *LOC108638451* |  |
| **snp51448-scaffold754-481947** | 21 | 20834768 | *ZNF710, IDH2, LOC102186633, SEMA4B, CIB1, GDPGP1, NGRN* |  |
| **snp57122-scaffold91-477178** | 21 | 4049239 | *-* | *GABRA5, LOC102178769* |
| snp57168-scaffold91-2506997 | 21 | 2019420 | *ATP10A, LOC102191179* |  |
| **snp57235-scaffold911-1204549** | 21 | 15959450 | *LOC106503345, KLHL25* |  |
| **snp7146-scaffold1265-72004** | 21 | 17693088 | *TRNAG-UCC* |  |
| **snp7153-scaffold1265-368894** | 21 | 17396198 | *-* | *AGBL1, TRNAG-UCC* |
| **snp2949-scaffold109-1754220** | 22 | 8404395 | *-* | *TRNAS-GGA, TRNAQ-CUG* |
| **snp11458-scaffold1417-946413** | 23 | 16256899 | *FARS2, LYRM4, LOC102169818* |  |
| snp20296-scaffold2009-4422 | 23 | 48440896 | *LOC102180545, KHDRBS2* |  |
| **snp39678-scaffold505-474676** | 23 | 36272066 | *DNAH8, GLO1, BTBD9* |  |
| snp47830-scaffold673-1651381 | 23 | 13185358 | *CDKAL1* |  |
| **snp10677-scaffold1379-313938** | 24 | 12707725 | *LOC102170373, SYT4* |  |
| snp22746-scaffold2267-268082 | 24 | 9175615 | *-* | *TMX3, DSEL* |
| **snp54916-scaffold84-1663231** | 24 | 3258805 | *-* | *ZNF516, TSHZ1* |
| **snp7659-scaffold1277-264730** | 24 | 49457980 | *LOC108633809, LIPG* | *LIPG, LOC108633809* |
| **snp9699-scaffold1349-495354** | 24 | 5803577 | *LOC102180814* |  |
| **snp38343-scaffold485-862961** | 25 | 16554591 | *CLEC19A, TRNAK-CUU, SYT17* |  |
| **snp43024-scaffold570-1172048** | 25 | 6941744 | *-* | *RBFOX1, TMEM114* |
| snp44155-scaffold6-1365946 | 25 | 26616447 | *SEPHS2, TRNAR-UCG,ITGAL, LOC102171833, ZNF68, LOC102172404, LOC102172682, PRR14, FBRS* |  |
| **snp15063-scaffold161-373737** | 26 | 14520936 | *KCNK18, VAX1, SHTN1* |  |
| **snp31449-scaffold3488-54845** | 26 | 48980676 | *ZWINT* |  |
| snp39726-scaffold508-144993 | 26 | 10036024 | *TACC2, NSMCE4A* |  |
| **snp42278-scaffold556-861145** | 26 | 8347596 | *CHST15, CPXM2* |  |
| snp52207-scaffold774-160624 | 26 | 42635096 | *SGMS1* |  |
| **snp55159-scaffold847-2003062** | 26 | 6080084 | *C26H10orf90, ADAM12* |  |
| **snp30300-scaffold333-3618181** | 27 | 9466438 | *SFRP1, LOC102185808* |  |
| **snp30734-scaffold34-1571279** | 27 | 3219984 | *LOC102174361* |  |
| **snp44450-scaffold604-977126** | 27 | 11448725 | *LOC102191489, ADAM9, LOC102191206* |  |
| **snp44483-scaffold604-2371901** | 27 | 12843500 | *-* | *TRNAE-UUC, LOC102185144* |
| **snp44504-scaffold604-3249879** | 27 | 13721478 | *-* | *KCNU1, LOC102184589* |
| snp51902-scaffold762-3218815 | 27 | 40863322 | *CSMD1* |  |
| **snp54865-scaffold838-4305667** | 28 | 16059681 | *USP54, LOC102187373, FUT11, SEC24C, SYNPO2L, MYOZ1, PPP3CB* |  |
| **snp57597-scaffold922-153439** | 28 | 546413 | *-* | *LOC102169834, CXCL12* |
| snp15245-scaffold1624-467976 | 29 | 41084398 | *LOC102180194, LOC102180465, LOC106503745, ASRGL1, AHNAK* |  |
| **snp17567-scaffold182-274423** | 29 | 43971595 | *POLA2, CDC43EP2, DPF2, TIGD3, SLC25A45, FRMD8, TRNAS-GGA* |  |
| **snp227-scaffold1006-227193** | 29 | 7986537 | *TMEM135, LOC102191495, FZD4* |  |
| **snp48271-scaffold683-1146836** | 29 | 11630471 | *DLG2* |  |
| **snp53108-scaffold799-3285503** | 29 | 35315323 | *TMEM45B, NFRKB, PRDM10* |  |
