# Supplementary material for: Analysis of genome-wide DNA arrays reveals the genomic population structure and diversity in autochthonous Greek goat breeds
Source: PLoS One. 2019 Dec 12;14(12):e0226179. doi: 10.1371/journal.pone.0226179 (PMC6907847; doi:10.1371/journal.pone.0226179)
Supplement: S7 Table — A.P.: Assignment Probability, SNPs: Single nucleotide polymorphisms. (DOCX) [file pone.0226179.s017.docx]

**S7 Table. Assignment probabilities for each individual using the 95 SNPs, calculated with the GeneClass 2 software.** A.P.: Assignment Probability, SNPs: Single Nucleotide Polymorphisms.

|  |  | Assign / exclude population as origin of individuals | | | | Detection of first generation migrants, likelihood computation (L=L_home/L_max) | | | |
| --- | --- | --- | --- | --- | --- | --- | --- | --- | --- |
|  |  | Frequency based method | | Bayesian method | | Frequency based method | | Bayesian method | |
|  |  | Correctly assigned: 100.0 % (64) | | Correctly assigned: 100.0 % (64) | | 0 individuals with a probability below 0.001 | | 0 individuals with a probability below 0.001 | |
| ID | Breed | Skopelos A.P. | Eghoria A.P. | Skopelos A.P. | Eghoria A.P. | -log(L) (Skopelos) | -log(L) (Eghoria) | -log(L) (Skopelos) | -log(L) (Eghoria) |
| EG1 | Eghoria | 0.000 | 0.652 | 0.000 | 0.907 | 123.783 | 34.494 | 117.758 | 34.537 |
| EG2 | Eghoria | 0.000 | 0.952 | 0.000 | 0.992 | 136.388 | 31.455 | 129.584 | 31.577 |
| EG3 | Eghoria | 0.000 | 0.342 | 0.000 | 0.531 | 92.148 | 38.001 | 88.653 | 37.972 |
| EG4 | Eghoria | 0.000 | 0.969 | 0.000 | 0.987 | 120.088 | 30.732 | 114.560 | 30.910 |
| EG5 | Eghoria | 0.000 | 0.896 | 0.000 | 0.979 | 137.859 | 32.330 | 131.116 | 32.388 |
| EG6 | Eghoria | 0.000 | 0.930 | 0.000 | 0.980 | 130.892 | 31.385 | 124.363 | 31.502 |
| EG7 | Eghoria | 0.000 | 0.956 | 0.000 | 0.999 | 128.072 | 30.891 | 122.323 | 31.054 |
| EG8 | Eghoria | 0.000 | 0.761 | 0.000 | 0.969 | 126.870 | 33.617 | 120.637 | 33.698 |
| EG9 | Eghoria | 0.000 | 0.958 | 0.000 | 0.999 | 130.270 | 31.228 | 124.078 | 31.377 |
| EG10 | Eghoria | 0.000 | 0.893 | 0.000 | 0.958 | 133.569 | 32.291 | 127.095 | 32.349 |
| EG11 | Eghoria | 0.000 | 0.957 | 0.000 | 0.990 | 130.748 | 30.775 | 124.425 | 30.933 |
| EG12 | Eghoria | 0.000 | 0.819 | 0.000 | 0.929 | 123.714 | 31.978 | 117.662 | 32.072 |
| EG13 | Eghoria | 0.000 | 0.842 | 0.000 | 0.958 | 130.738 | 33.040 | 123.965 | 33.121 |
| EG14 | Eghoria | 0.000 | 0.948 | 0.000 | 0.991 | 131.523 | 31.507 | 124.998 | 31.658 |
| EG15 | Eghoria | 0.000 | 0.049 | 0.000 | 0.145 | 63.416 | 46.704 | 61.607 | 46.391 |
| EG16 | Eghoria | 0.000 | 0.200 | 0.000 | 0.282 | 76.242 | 41.748 | 73.832 | 41.627 |
| EG17 | Eghoria | 0.000 | 0.067 | 0.000 | 0.176 | 71.718 | 46.891 | 69.682 | 46.539 |
| EG18 | Eghoria | 0.000 | 0.308 | 0.000 | 0.512 | 81.191 | 40.146 | 78.583 | 40.082 |
| EG19 | Eghoria | 0.000 | 0.064 | 0.000 | 0.200 | 81.759 | 46.473 | 78.880 | 46.148 |
| EG20 | Eghoria | 0.000 | 0.043 | 0.000 | 0.156 | 66.501 | 47.723 | 64.574 | 47.370 |
| EG21 | Eghoria | 0.000 | 0.147 | 0.000 | 0.314 | 88.263 | 43.721 | 85.229 | 43.463 |
| EG22 | Eghoria | 0.000 | 0.188 | 0.000 | 0.300 | 74.571 | 41.414 | 72.347 | 41.291 |
| EG23 | Eghoria | 0.000 | 0.246 | 0.000 | 0.439 | 88.059 | 41.257 | 85.020 | 41.102 |
| EG24 | Eghoria | 0.000 | 0.010 | 0.000 | 0.076 | 61.794 | 48.446 | 60.023 | 48.008 |
| EG25 | Eghoria | 0.000 | 0.216 | 0.000 | 0.427 | 83.775 | 42.076 | 81.086 | 41.885 |
| EG26 | Eghoria | 0.000 | 0.372 | 0.000 | 0.662 | 91.677 | 37.137 | 88.176 | 37.183 |
| EG27 | Eghoria | 0.000 | 0.304 | 0.000 | 0.463 | 85.475 | 40.373 | 82.299 | 40.316 |
| EG28 | Eghoria | 0.000 | 0.231 | 0.000 | 0.426 | 88.059 | 41.257 | 85.020 | 41.102 |
| EG29 | Eghoria | 0.000 | 0.050 | 0.000 | 0.164 | 78.207 | 47.182 | 75.418 | 46.773 |
| EG30 | Eghoria | 0.000 | 0.158 | 0.000 | 0.216 | 67.131 | 41.229 | 65.344 | 41.069 |
| EG31 | Eghoria | 0.000 | 0.311 | 0.000 | 0.487 | 88.080 | 39.668 | 85.170 | 39.550 |
| EG32 | Eghoria | 0.000 | 0.126 | 0.000 | 0.264 | 77.163 | 43.705 | 74.834 | 43.438 |
| SK1 | Skopelos | 0.696 | 0.000 | 0.926 | 0.000 | 25.291 | 84.032 | 25.326 | 82.066 |
| SK2 | Skopelos | 0.875 | 0.000 | 0.987 | 0.000 | 23.655 | 89.044 | 23.787 | 86.921 |
| SK3 | Skopelos | 0.679 | 0.000 | 0.917 | 0.000 | 25.799 | 80.380 | 25.919 | 78.652 |
| SK4 | Skopelos | 0.658 | 0.000 | 0.853 | 0.000 | 24.849 | 78.553 | 24.902 | 76.817 |
| SK5 | Skopelos | 0.611 | 0.000 | 0.857 | 0.000 | 26.442 | 87.469 | 26.446 | 85.413 |
| SK6 | Skopelos | 0.920 | 0.000 | 0.971 | 0.000 | 23.162 | 84.193 | 23.348 | 82.281 |
| SK7 | Skopelos | 0.856 | 0.000 | 0.983 | 0.000 | 23.503 | 87.932 | 23.654 | 85.750 |
| SK8 | Skopelos | 0.792 | 0.000 | 0.934 | 0.000 | 23.807 | 83.036 | 23.927 | 81.111 |
| SK9 | Skopelos | 0.815 | 0.000 | 0.970 | 0.000 | 24.492 | 86.180 | 24.626 | 84.149 |
| SK10 | Skopelos | 0.518 | 0.000 | 0.730 | 0.000 | 27.064 | 85.879 | 27.286 | 83.724 |
| SK11 | Skopelos | 0.070 | 0.000 | 0.194 | 0.001 | 34.596 | 74.757 | 34.499 | 73.249 |
| SK12 | Skopelos | 0.679 | 0.000 | 0.873 | 0.000 | 25.326 | 83.970 | 25.321 | 82.028 |
| SK13 | Skopelos | 0.209 | 0.000 | 0.415 | 0.000 | 31.053 | 74.574 | 30.948 | 72.981 |
| SK14 | Skopelos | 0.006 | 0.000 | 0.037 | 0.000 | 39.476 | 69.908 | 38.924 | 68.569 |
| SK15 | Skopelos | 0.302 | 0.000 | 0.557 | 0.000 | 29.603 | 74.016 | 29.567 | 72.545 |
| SK16 | Skopelos | 0.070 | 0.000 | 0.124 | 0.000 | 35.432 | 74.016 | 35.062 | 72.476 |
| SK17 | Skopelos | 0.152 | 0.000 | 0.397 | 0.000 | 32.380 | 77.547 | 32.076 | 75.896 |
| SK18 | Skopelos | 0.241 | 0.000 | 0.454 | 0.000 | 31.145 | 76.247 | 31.000 | 74.732 |
| SK19 | Skopelos | 0.768 | 0.000 | 0.953 | 0.000 | 24.611 | 83.506 | 24.711 | 81.635 |
| SK20 | Skopelos | 0.005 | 0.000 | 0.020 | 0.000 | 40.142 | 69.885 | 39.646 | 68.629 |
| SK29 | Skopelos | 0.026 | 0.000 | 0.083 | 0.001 | 37.134 | 66.809 | 36.815 | 65.730 |
| SK30 | Skopelos | 0.664 | 0.000 | 0.876 | 0.000 | 25.614 | 80.721 | 25.737 | 78.934 |
| SK31 | Skopelos | 0.115 | 0.000 | 0.258 | 0.000 | 32.250 | 79.483 | 31.842 | 77.682 |
| SK32 | Skopelos | 0.983 | 0.000 | 0.999 | 0.000 | 21.078 | 85.120 | 21.358 | 83.207 |
| SK33 | Skopelos | 0.085 | 0.000 | 0.260 | 0.001 | 34.023 | 74.733 | 33.844 | 73.166 |
| SK34 | Skopelos | 0.963 | 0.000 | 1.000 | 0.000 | 21.681 | 88.043 | 21.889 | 85.940 |
| SK35 | Skopelos | 0.054 | 0.000 | 0.158 | 0.001 | 35.855 | 71.826 | 35.541 | 70.507 |
| SK36 | Skopelos | 0.773 | 0.000 | 0.960 | 0.000 | 24.656 | 81.997 | 24.804 | 80.200 |
| SK37 | Skopelos | 0.314 | 0.000 | 0.513 | 0.000 | 29.526 | 72.213 | 29.416 | 70.922 |
| SK38 | Skopelos | 0.621 | 0.000 | 0.890 | 0.000 | 26.308 | 86.917 | 26.166 | 84.794 |
| SK39 | Skopelos | 0.878 | 0.000 | 0.991 | 0.000 | 23.554 | 87.950 | 23.712 | 85.842 |
| SK40 | Skopelos | 0.306 | 0.000 | 0.545 | 0.000 | 30.193 | 79.140 | 30.020 | 77.454 |
